# The UPR sensor IRE1α and the adenovirus E3-19K glycoprotein sustain persistent and lytic infections

**DOI:** 10.1038/s41467-020-15844-2

**Published:** 2020-04-24

**Authors:** Vibhu Prasad, Maarit Suomalainen, Yllza Jasiqi, Silvio Hemmi, Patrick Hearing, Louise Hosie, Hans-Gerhard Burgert, Urs F. Greber

**Affiliations:** 1 Department of Molecular Life Sciences, University of Zurich, Zurich, Switzerland; 2 Department of Molecular Genetics and Microbiology, Renaissance School of Medicine, Stony Brook University, Stony Brook, NY USA; 3 University of Warwick, School of Life Sciences, Coventry, CV4 7AL UK; 4 Present Address: The Francis Crick Institute, 1 Midland Road, London, NW1 1AT UK; 5 Present Address: Institute of Virology, University Medical Center Freiburg, 79104 Freiburg, Germany

**Keywords:** Mechanisms of disease, Adenovirus

## Abstract

Persistent viruses cause chronic disease, and threaten the lives of immunosuppressed individuals. Here, we elucidate a mechanism supporting the persistence of human adenovirus (AdV), a virus that can kill immunosuppressed patients. Cell biological analyses, genetics and chemical interference demonstrate that one of five AdV membrane proteins, the E3-19K glycoprotein specifically triggers the unfolded protein response (UPR) sensor IRE1α in the endoplasmic reticulum (ER), but not other UPR sensors, such as protein kinase R-like ER kinase (PERK) and activating transcription factor 6 (ATF6). The E3-19K lumenal domain activates the IRE1α nuclease, which initiates mRNA splicing of X-box binding protein-1 (XBP1). XBP1s binds to the viral E1A-enhancer/promoter sequence, and boosts E1A transcription, E3-19K levels and lytic infection. Inhibition of IRE1α nuclease interrupts the five components feedforward loop, E1A, E3-19K, IRE1α, XBP1s, E1A enhancer/promoter. This loop sustains persistent infection in the presence of the immune activator interferon, and lytic infection in the absence of interferon.

## Abstract (web summary)

Adenovirus (AdV) can cause persistent infections, but underlying mechanisms are poorly understood. Here, Prasad et al. show that the AdV glycoprotein E3-19K activates the unfolded protein response sensor IRE1α, and that this triggers a feedforward loop that sustains persistent infection in the presence of interferon.

## Introduction

Pathogens persist within host cells, which makes them difficult to treat as they minimally interfere with the host and escape immune clearance. Viral persistence comprises chronic and latent infections, for example, human immunodeficiency virus (HIV), herpesvirus, papilloma virus, polyoma virus, hepatitis B virus, and human adenovirus (AdV)^1–3^. Viral persistence involves the restoration of homeostasis upon initial infection. The unfolded protein response (UPR) restores homeostasis upon ER stress. Enveloped viruses, including influenza virus and herpes viruses subvert the UPR for the synthesis of viral glycoproteins^4–6^. They involve three transmembrane sensors in the ER, the inositol-requiring enzyme 1 (IRE1), activating transcription factor 6 (ATF6/p90), and protein kinase RNA-like ER kinase (PERK). IRE1α, ATF6, and PERK enhance the levels of the transcription factors XBP1s, ATF6/p50, and ATF4, respectively, and upregulate gene expression of chaperones and slow down protein translation restoring ER homeostasis^7–10^. IRE1α has cytoplasmic kinase and nuclease domains. Its phosphorylation enhances ATP binding, activates the ribonuclease, and initiates the splicing of XBP1 mRNA by removing a short intron of 26 nucleotides (nt)^11^. Back-ligation of the spliced mRNA by the tRNA ligase Rtcb generates XBP1s mRNA encoding the functional transcription factor XBP1s^12^. During the lytic cycle, Kaposi sarcoma herpesvirus activates all three UPR sensors, and inhibits the downstream transcription of host genes by yet unknown mechanisms reviewed in ref. ^13^.

Here, we explored how the UPR affected the nonenveloped non-enveloped AdV. AdVs are widespread pathogens causing self-limiting disease in the eyes (species B, D, and E), the upper and lower respiratory tracts (species B, C, and E), and the gastrointestinal tract (species F and G)^14^. AdV-B, C, E, and F persist in lymphoid and other cell types of the digestive tracts, and produce low levels of particles in the presence of cellular and humoral cytokines, interferon (IFN) type I, or the proinflammatory IFN-γ^15–18^. In immunocompromised or stressed individuals, AdVs give rise to morbidity and mortality^19,20^.

AdV enters cells by receptor-mediated endocytosis, endosomal rupture, and cytoplasmic transport, and dismantles the capsid to deliver viral DNA (vDNA) into the nucleus for progeny production and establishment of persistence^21–23^. Expression of the immediate early viral *E1A* gene gives rise to transactivators from alternatively spliced mRNA^24,25^. E1A proteins interact with numerous host proteins on double-stranded DNA, and control host transcription, the cell cycle, DNA replication, and suppress the expression of IFN-stimulated genes. They activate all early AdV promoters, including those controlling *E1B, E2, E3,* and *E4*, and drive viral replication^21,26,27^.

The *E3* transcription unit protects the host from disease, especially uncontrolled inflammatory response, and the virus from eradication^28–31^. AdV-C *E3* encodes seven open-reading frames (ORFs), five of which are membrane proteins. The *E3A* region gives rise to CR1-alpha, also known as 7.1K (AdV-C5) or 6.7K (AdV-C2), which inhibit apoptosis by degrading the TNF-related apoptosis-inducing ligand (TRAIL) receptor 2. The glycoprotein E3-19K (short 19K) and the AdV death protein (ADP in AdV-C) of *E3A* suppress cellular immune responses and may promote lytic virus release, respectively^29^. 19K suppresses the adaptive immune response toward AdV-infected cells by blocking the transport of MHC class-I molecules from the ER to the cell surface, and reduces the activation of cytotoxic CD8 T cells and NK cells^32–35^. The *E3B* region encodes RIDα and RIDβ blocking apoptosis mediated through tumor necrosis factor (TNF), Fas ligand, and TRAIL signaling.

Recent studies with telomerase reverse transcriptase (TERT)-immortalized human dermal fibroblasts established a persistence model for AdV, where IFN-I or IFN-II suppresses AdV-C5 infection by reducing the recruitment of the positive transcription regulator GABPα/β, and enhancing the E2F/Rb repressor complex on the *E1A* promoter sequence^36^. Removal of IFN leads to virus lytic release, akin to acutely immunosuppressed patients^17^. Here, we show that the AdV glycoprotein 19K selectively activates IRE1α but not PERK and ATF6. This gives rise to a transcriptional feedforward loop, including five components—19K, IRE1α, XBP1s, the *E1A* enhancer/promoter (e/p), and E1A protein. This loop maintains long-term viral persistence in the presence of IFN, and boosts lytic infection in the absence of IFN.

## Results

### IRE1α-mediated XBP1 splicing enhances AdV infection

**Fig. 1:**
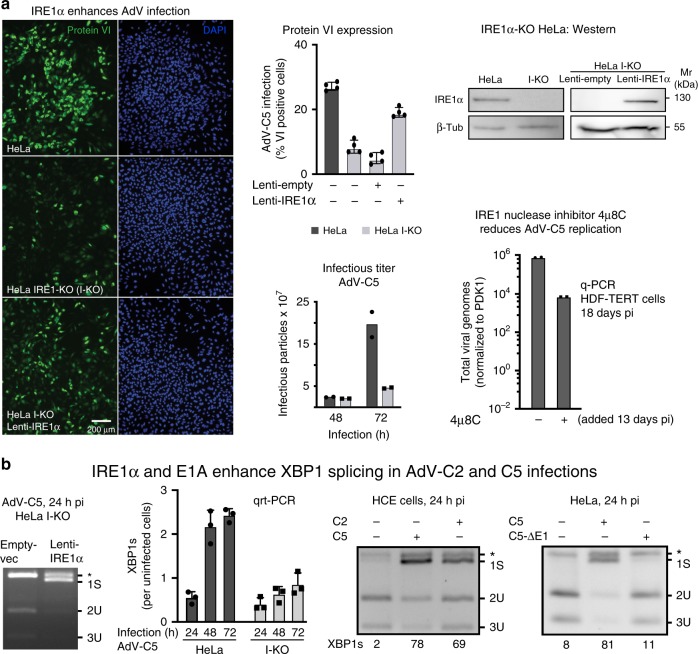
IRE1α activation enhances AdV infection of HeLa cells. **a** IRE1α-knockout (I-KO) HeLa cells are less susceptible to infection by AdV-C5 (MOI 75, 75 vp/cell) compared with normal HeLa, as indicated by late viral protein VI expression, whereas the ectopic lentivirus-mediated expression of IRE1α in HeLa I-KO cells restores infection (left panel, upper row middle and right panels showing representative images and quantifications, respectively. Scale bar, 200 µm). Data show the means ± SD from four independent experiments. Reduced virus growth in HeLa I-KO cells compared with wild-type cells. Cells were infected with AdV-C5 (MOI 500) for 1 h at 37 °C (equivalent to MOI 50 in continuous infection), unbound virus washed off, and virus titers from cells and supernatant measured at 48 and 72 hpi (lower row middle panel). Data show the means from two independent experiments. The IRE1α endonuclease inhibitor 4µ8C reduces AdV-C5 titers in long-term infections of HDF-TERT cells (lower row, right panel). HDF-TERT cells were infected with AdV-C5 (MOI 200, 37 °C, 1 h), followed by addition of 4µ8C (100 µM) 13 days pi. Data show the means from two independent experiments. **b**. IRE1α and the expression of the viral E1A protein are required to enhance XBP1 splicing in AdV-C2 and C5 infections. Rescue of XBP1 splicing in AdV-infected I-KO cells by IRE1α overexpression (first panel). Cells were transduced and infected as in **a**; cell lysates were subjected to XBP1 splicing assays at 24 hpi. Reduced XBP1s transcripts in HeLa I-KO compared with normal HeLa cells upon AdV-C5 infection (MOI 5, second panel). Data show the means ± SD from three independent experiments. XBP1 splicing in human conjunctival epithelial cells infected with AdV-C2 or C5 (MOI 75, third panel). *E1A*-deleted AdV-C5 mutant does not activate XBP1 splicing in HeLa cells 24 hpi (MOI 200 each, fourth panel). The asterisk denotes a background product. Source data are provided as a Source Data file.

Mammalian cells express two homologs of the yeast Ire1p, IRE1α (encoded by the ERN1 gene) and IRE1β (ERN2), the latter in a tissue-specific manner, for example in the digestive tract^37,38^. To explore the role of IRE1α in AdV infection, we used CRISPR/Cas9 to generate IRE1α-knockout HeLa cells (HeLa I-KO). A guide RNA targeting exon 2 yielded a KO phenotype affecting all three allelic copies of IRE1α (Fig. 1a, Supplementary Fig. 1a, b). Two IRE1α alleles were edited by frameshift mutations and one had a 15-nt in-frame deletion. HeLa I-KO was significantly less susceptible to AdV-C5 infection than HeLa or HeLa I-KO ectopically expressing IRE1α from a lentivirus, as shown by AdV late protein VI expression and virus production (Fig. 1a). Infection inhibition was not due to reduced virus entry, as the incoming vDNA was effectively delivered into the nucleus 2.5 hpi^39^ (Supplementary Fig. 1c). Akin to IRE1α KO, the IRE1α nuclease inhibitor 4µ8C^40^ reduced viral replication, as shown by quantitative (q)-PCR (Fig. 1a).

To test if AdV infection enhanced the IRE1α nuclease activity, we measured the levels of XBP1s by quantitative reverse transcriptase (qrt) PCR in extracts of infected HeLa and HeLa I-KO cells^41^. AdV-C5 infection consistently increased the XBP1s mRNA levels at 24, 48, or 72 hpi (Fig. 1b). Note that the band denoted with asterisk (*) is a background product and can be removed by EndoT digest (see Supplementary Fig. 1d). XBP1s (denoted as 1S) was not enhanced in HeLa I-KO cells, but restored by ectopic expression of IRE1α (Fig. 1b). Importantly, XBP1s induction occurred in AdV-C2 or C5-infected HeLa and human corneal epithelial (HCE) cells, or in diploid human fibroblasts WI-38, but not in cells infected with AdV-C5 lacking the E1 region (Fig. 1b, Supplementary Fig. 1e–g). E1 encodes the E1A immediate early transactivator protein, the antiapoptotic E1B-19K and E1B-55K proteins^24,42^. Collectively, the data show that AdV infection of both transformed and nontransformed non-transformed human cells induces XBP1s depending on IRE1α and the early viral genes *E1A–E1B*.

### Phosphorylation of IRE1α and not PERK in AdV infection

**Fig. 2:**
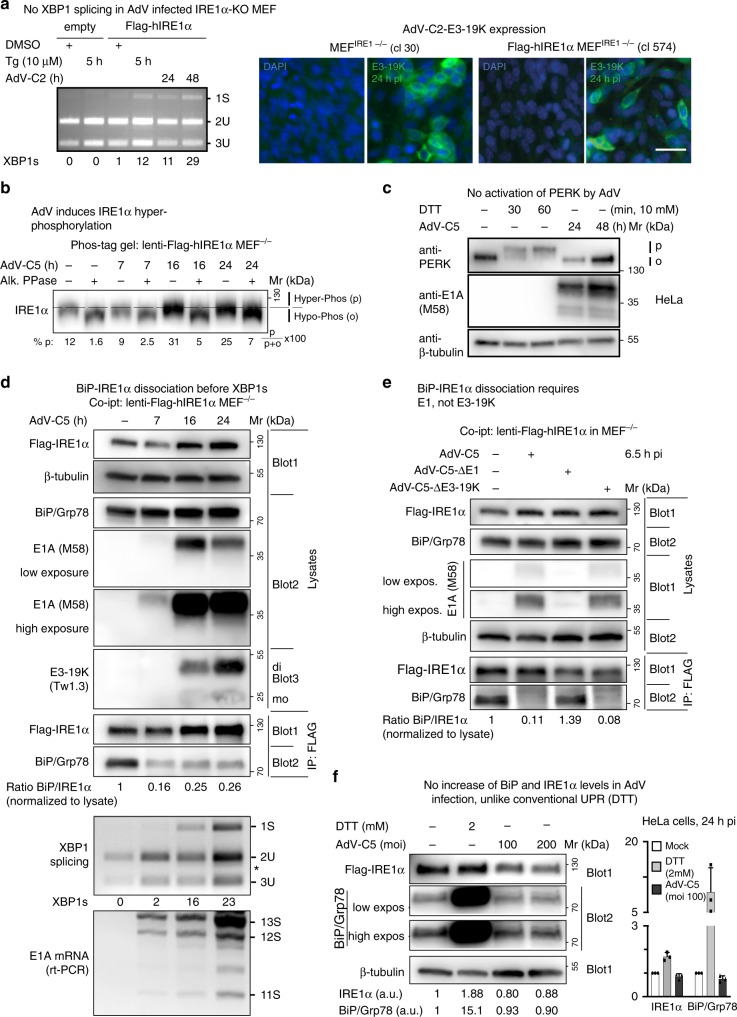
AdV infection of mouse embryonic fibroblasts induces XBP1 splicing and displaces BiP/Grp78 from IRE1α. **a** Flag-IRE1α-expressing MEFs but not IRE1α-KO MEFs induce XBP1s upon AdV-C2 infection (MOI 300) or thapsigargin (Tg, 10 µM) treatment (left panel). Representative immunofluorescence images with the anti-19K antibody 3A9 showing 19K expression in AdV-C-infected IRE1α-KO MEFs and Flag-IRE1α-expressing IRE1α-KO MEFs 24 hpi (clones 30 and 574, respectively, right panel, scale bar, 20 µm). Three independent experiments gave similar results. Source data are provided as a Source Data file. **b** AdV induces phosphorylation of IRE1α. Flag-IRE1α expressing IRE1α-KO MEFs at 7, 16, and 24 hpi with AdV-C5 (MOI 300) immunoblotted with anti-IRE1α antibody. Lysates were resolved on a 6% SDS-PAGE gel containing 25 µM Phos-tag. Samples were treated with or without alkaline phosphatase. Phosphorylated (p) and hypophosphorylated (o) forms of IRE1α are indicated by the dashed lines, and the percentage of IRE1α phosphorylated was calculated as indicated. Lysates are the same as in panel D demonstrating β-tubulin loading. Three independent experiments gave similar results. Source data are provided as a Source Data file. **c** AdV-C5 infection of HeLa cells (MOI 200) does not activate PERK, unlike treatment of cells with the reducing agent DTT. Activated phosphorylated PERK is indicated by p, and the inactive form by o. Two independent experiments gave similar results. **d** BiP displacement from IRE1α occurs before XBP1 splicing in Flag-IRE1α-expressing IRE1α-KO MEFs infected with AdV-C5 (MOI 300). Cells were lysed and BiP–IRE1α complexes immunoprecipitated (IP) with anti-Flag antibody, and a western blot with anti-IRE1α, anti-BiP, and anti-β-tubulin antibodies was performed. A separate non-reducing immunoblot probed with anti-19K Tw1.3 antibodies revealed monomeric and dimer forms of 19K indicated as mo and di, respectively. Input lysates were 1% of the immunoprecipitated samples. The corresponding samples were also analyzed for XBP1 splicing and E1A mRNA levels by rt-PCR (reverse transcription polymerase chain reaction), as indicated. Three independent experiments gave similar results. Source data are provided as a Source Data file. **e** BiP–IRE1α dissociation requires E1, not 19K. Co-immunoprecipitation of Flag-IRE1α and BiP was performed as described in **d** with AdV mutants lacking E1 (AdV-C5-∆E1) and 19K (AdV-C5-Δ19K) at MOI 300 each. Two independent experiments gave similar results. Source data are provided as a Source Data file. **f** AdV-C5 infection does not increase BiP/Grp78 and IRE1α levels given in arbitrary units (a.u.), unlike the canonical UPR triggered by DTT (2 mM). The bar graph shows the normalized levels of IRE1α and BiP from three independent experiments. Data show the means ± SD from three independent experiments. Source data are provided as a Source Data file.

We next tested if XBP1s induction was conserved in murine cells. AdV-C5 infection for 24–48 h readily induced XBP1s in mouse embryonic fibroblasts (MEFs) expressing Flag-IRE1α, but not in cells lacking IRE1α (I-KO)^43^, akin to the short-term chemical stimulation of ER stress by thapsigargin (5 h), which inhibits the ER calcium pump and depletes calcium ions from ER stores^44^ (Fig. 2a). Both I-KO and normal MEFs were readily infected with AdV-C2, as indicated by the expression of the early 19K glycoprotein.

To test if AdV-C5 activated IRE1α, we analyzed the migration of the endogenous IRE1α protein in SDS-PAGE containing Phos-Tag. Such gels retard the migration of phosphoproteins relative to the nonphosphoproteins. IRE1α migration was retarded upon AdV-C5 infection at 16 or 24 hpi, and retardation blunted by treatment of the cell lysates with phosphatase (Fig. 2b). In contrast, PERK did not show an upward shift upon AdV-C5 infection, unlike treatment with the reducing agent dithiothreitol (DTT), a known activator of PERK^45^ (Fig. 2c). We found no evidence that AdV-C5 induced the regulated IRE1α-dependent decay (RIDD) pathway, unlike DTT, as assayed with Bloc1S1 mRNA (see Supplementary Fig. 2a).

### BiP dissociates from IRE1α in AdV infection independent of 19K

In UPR, the association of the ER chaperone BiP/Grp78 (BiP) with IRE1α decreases upon initial ER stress, and restores under persistent UPR stimulation, when BiP and co-chaperones are transcriptionally induced^46,47^. We assessed the levels of BiP–IRE1α by immunoprecipitation experiments of Flag-hIRE1α expressed in IRE1α^−/−^ MEFs at near-endogenous levels^43^. The co-immunoprecipitation data, western blots, and rt-PCR measurements of E1A mRNA showed that BiP dissociated from IRE1α at 7 hpi, when only low amounts of E1A protein were present, and neither 19K protein nor XBP1 splicing were detectable (Fig. 2d). This was in contrast to conventional UPR induced by DTT where BiP–IRE1α dissociation was rapidly followed by XBP1 splicing (Supplementary Fig. 2b). Similar results were obtained in HeLa I-KO cells transduced with lentivirus encoding Flag-hIRE1α, where BiP dissociated from IRE1α at 4 h post AdV-C2 infection (Supplementary Fig. 2c, d). In MEFs, AdV-C5 lacking *E1A* and *E1B* ORFs did not dissociate BiP from IRE1α, in contrast to a mutant lacking the 19K ORF (Fig. 2e). Notably, AdV-C5 infection did not increase the levels of BiP and IRE1α, unlike treatment with DTT, which massively increased the BiP levels (Fig. 2f). Accordingly, the ectopic expression of BiP did not attenuate the induction of XBP1s by AdV-C2, unlike thapsigargin treatment (Supplementary Fig. 2e, f), consistent with earlier reports^46^. Collectively, the data indicate that AdV activates IRE1α in a non-canonical manner, whereby BiP dissociates long before XBP1 splicing, and independent of 19K.

### An early AdV gene product activates IRE1α

E1A activates all early viral promoters, and together with E1B enhances transformation and replication^48,49^. We assessed if viral replication was required for the IRE1α/XBP1s induction by treating HeLa cells with the nucleoside analog cytosine arabinoside (AraC), which blocks viral replication and late protein expression past the immediate early phase of *E1A* induction^50,51^. AraC neither affected the *E1A* expression nor the levels of XBP1s (Supplementary Fig. 3a). In contrast, an inhibitor of the positive transcription elongation factor, flavopiridol, blocked *E1A* expression 24 hpi as expected^52^, and wiped out the induction of XBP1s (Supplementary Fig. 3b). The data reinforce the notion that E1A is required for XBP1s induction.

### The lumenal domain of C2/5–19K activates IRE1α

**Fig. 3:**
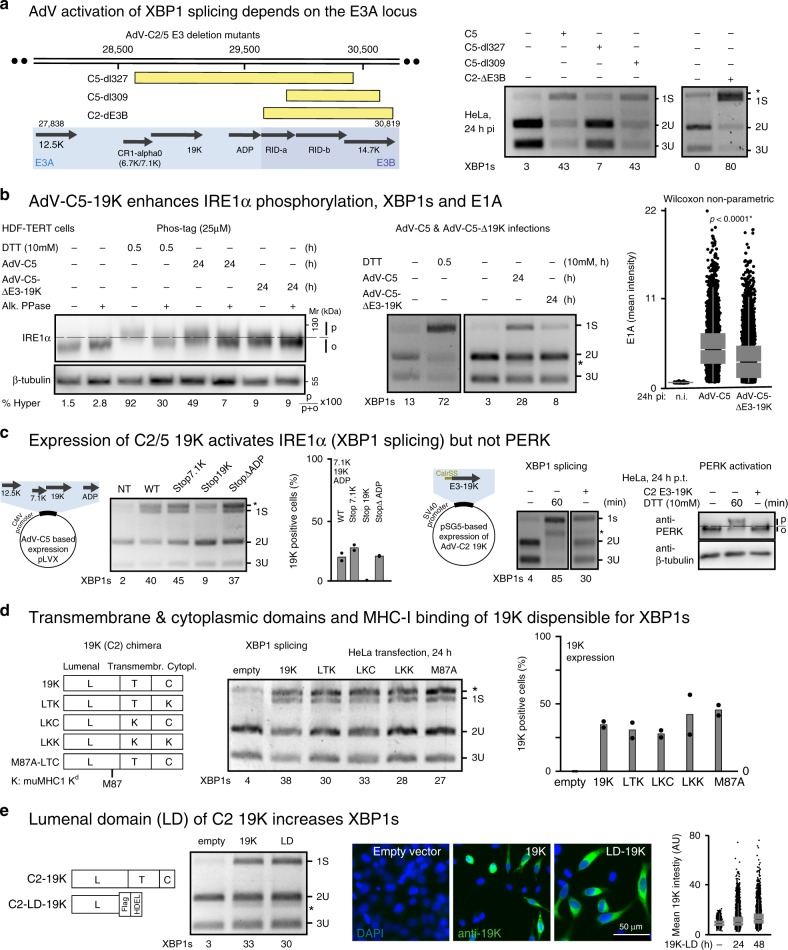
The lumenal domain of the 19K glycoprotein activates IRE1α. **a** Schematic drawing showing the deletions in the E3 region of AdV mutants with yellow boxes indicating the deletions (left panel). Infection was carried out with a median of 150 particles bound per cell. Asterisk denotes a background product. At least three independent experiments gave similar results. Source data are provided as a Source Data file. **b** AdV-C5 19K enhances IRE1α phosphorylation, XBP1s splicing, and E1A levels. Phosphorylation of IRE1α in AdV-C5- and AdV-C5-d19K-infected HDF-TERT cells (MOI 75000, 24 hpi) was analyzed in lysates treated with or without alkaline phosphatase, and fractionated by SDS-PAGE (6%, containing 25 µM Phos-tag, first panel). XBP1 splicing in AdV-C5- and AdV-C5-d19K-infected HDF-TERT cells 24 hpi (MOI 75,000, second panel). Immunofluorescence data of E1A are shown as a scatterplot using *n* = 14,000 cells randomly chosen per condition, 24 hpi (MOI 75000). Central line of the box plot indicates median with first and third quartiles, and whiskers are shown as boxes and lines, respectively. Statistics were performed using the Wilcoxon two-sided nonparametric test with *p < 0.0001 (third panel). Three independent experiments gave similar results. Source data are provided as a Source Data file. **c** Schematic depiction of lentiviral vectors expressing the AdV-C2 E3A locus open-reading frames (left panel). Premature termination of C2 E3A genes encoding the membrane proteins 7.1K, and ADP maintains XBP1s induction, whereas premature termination of 19K abolishes it (second panel). Data show the means from two technical replicates. Two independent experiments gave similar results. The western blot in the last panel displays activated phosphorylated (p) and unphosphorylated PERK (o). Source data are provided as a Source Data file. **d** Chimeric and mutant 19K constructs lacking transmembrane and cytoplasmic domains or the MHC-I binding site (M87A mutant) can induce XBP1s (left and middle panels). The wild-type lumenal, transmembrane, and cytoplasmic domains are labeled as L, T, and C, respectively, whereas mutant murine MHC-I domain replacing them is labeled as K^56^. The relative 19K expression levels in HeLa cells are shown in the right panel after Tw1.3 staining. Data are presented as mean from two technical replicates, and two independent experiments gave similar results. Source data are provided as a Source Data file. **e** Expression of the lumenal domain (LD) of C2 19K with a Flag tag, and a C-terminal ER retention signal (HDEL) is sufficient to induce XBP1s to levels similar as from the full-length 19K construct (left and middle panels). Immunofluorescence staining with the anti-19K 3A9 antibody in parallel samples depicts the expression levels of the full-length and the lumenal 19K proteins (right panel, scale bar, 50 µm). Data show a scatterplot of 20,000 randomly chosen cells per sample. Central line of the box plot indicates median with first and third quartiles, and whiskers are shown as boxes and lines, respectively. Source data are provided as a Source Data file.

We used a range of E3 mutant viruses to test for induction of XBP1s (Fig. 3a). AdV-C5-dl327 lacks *7.1K, 19K, ADP,* and *RIDα/β*^53^, dl309 lacks *RIDα/β* and *14.7K*^54^, and AdV-C2-dE3B has a deletion of *RIDα/β*^55^. The deletions in dl327 and dl309 were validated by PCR, and in addition, all three viruses were found to express E1A and 19K, except dl327 that lacked *19K* (Supplementary Fig. 3c). Importantly, dl327 did not activate IRE1α unlike dl309 and AdV-C2-dE3b (Fig. 3a).

We investigated the role of 19K in the activation of IRE1α in HDF-TERT cells. Phos-tag SDS-PAGE indicated that a mutant lacking the *19K* ORF (AdV-C5-Δ19K) poorly induced IRE1α phosphorylation, and barely induced XBP1s at 24 hpi unlike AdV-C5 (Fig. 3b). Similar results were obtained in time-course studies in HeLa cells (Supplementary Fig. 3d). Remarkably, the AdV-C5-Δ19K-infected cells showed reduced E1A expression levels compared with AdV-C5. This finding was in accordance with the RNAi data against 19K, where a pool of more than a dozen synthetic dsRNAs targeting 19K strongly reduced the induction of XBP1s, and also reduced the expression of E1A, while control RNAi pools against *E4-Orf4* had no effect on E1A (Supplementary Fig. 3e).

To test if 19K was sufficient for the induction of XBP1s, we coexpressed the AdV-C5 E3A ORFs *12.5K, 7.1K, ADP*, and *19K*. Together, they strongly induced XBP1s in HeLa cells (Fig. 3c). The abrogation of individual ORFs demonstrated that expression of 19K was necessary for XBP1s induction (Fig. 3c, Supplementary Fig. 3f). The expression of 19K alone was sufficient to induce XBP1s, but not PERK phosphorylation, unlike DTT (Fig. 3c).

The 19K glycoprotein has an N-terminal cleavable signal sequence, a transmembrane segment, and a small cytosolic tail with a di-lysine ER retention signal^56^. To identify the domain that triggered IRE1α activation, we expressed 19K chimeras with transmembrane and cytoplasmic domains from murine MHC-I H-2K^d^ in HeLa cells^35^. All three chimeras had comparable expression levels and induced XBP1s, but 19K lacking the LD only weakly induced XBP1s (Fig. 3d, and Supplementary Fig. 3g). The human MHC class-I antigen binding defective M87A 19K mutant^35^ strongly induced XBP1s, indicating that MHC-I binding was not required for XBP1s induction (Fig. 3d). The expression of the C2 19K-LD alone with or without a C-terminal HDEL motif activated IRE1α to comparable levels as the full-length protein, and much more effectively than the D8 19K-LD or full-length 19K (Fig. 3e, Supplementary Fig. 3h, i). The AdV-C2/5–19K glycoproteins have a highly conserved LD of 122 and 123 amino acids, 92% of which are identical, whereas the D8-LD of 119 amino acids is 30% identical with the C2/5-LDs (Supplementary Fig. 3j). This underscores that 19K alone or in context of AdV-C infection induces the phosphorylation of endogenous IRE1α followed by XBP1 splicing. This suggests the formation of IRE1α oligomers triggering *trans*-autophosphorylation and allosteric activation of the endonuclease domain^9^.

### The C2-19K lumenal domain interacts with IRE1α but not PERK

**Fig. 4:**
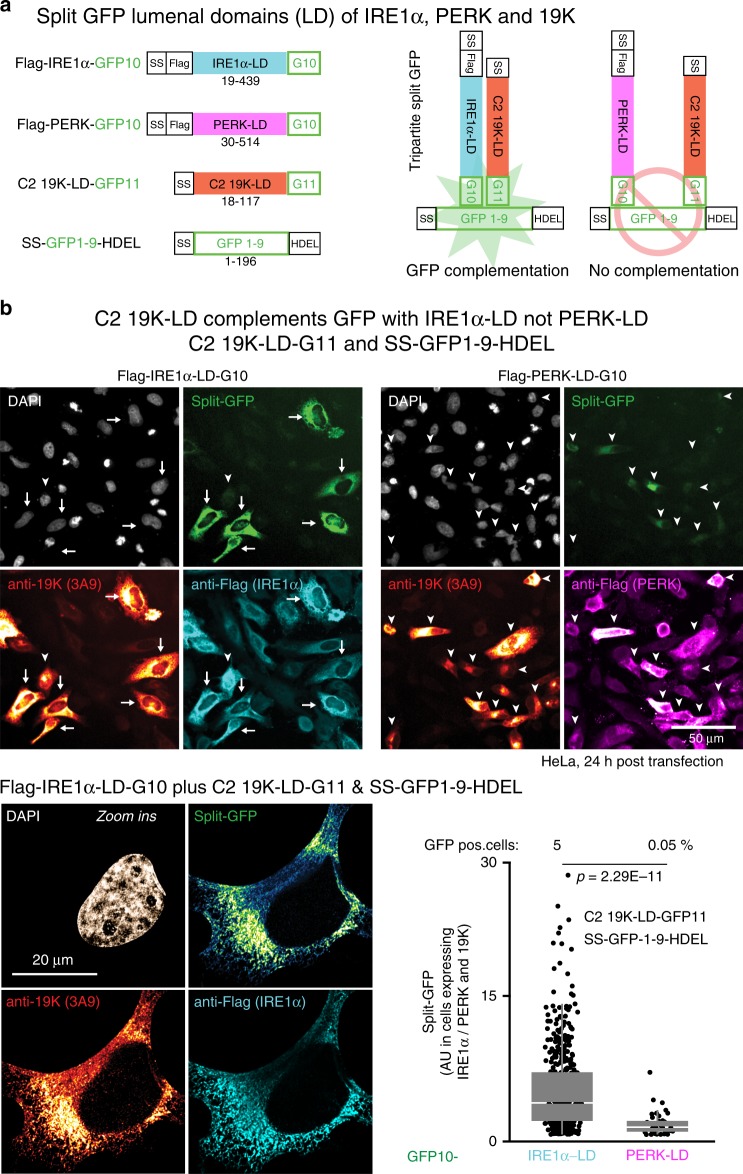
19K forms a complex with IRE1α in the ER. **a** Schematic representation of tripartite split-GFP lumenal domain (LD) constructs used in ER-lumenal GFP complementations. Green fluorescence is restored when proteins containing the GFP10 and GFP11 domains are in close proximity together with the core GFP1–9 targeted to the ER lumen. **b** Interaction of C2 19K-LD with IRE1α-LD but not PERK-LD. HeLa cells co-transfected with C2 19K-LD-GFP11, SS-(signal sequence)-GFP1–9-HDEL, and Flag-IRE1α-GFP10 or PERK-LD-GFP10 were fixed and stained with anti-19K (3A9) and anti-Flag antibodies and DAPI (nuclei). Confocal images were segmented with CellProfiler using DAPI as a nuclear mask, and the reticular ER signal was measured in a ten-pixel area around the nuclei. Arrows indicate high-intensity split-GFP complementation signals in IRE1α-LD-transfected cells, and arrowheads low-intensity split-GFP complementation. Zoomed in representative immunofluorescence micrographs of split-GFP complementation in SS-GFP1–9-HDEL, IRE1α-LD-GFP10, and C2 19K-LD-GFP11-transfected cells. Cells were imaged with Leica SP8 microscope using Nyquist x–y–z sampling with ×63 objective. Following acquisition, images were deconvolved using SVI Huygens using theoretical point-spread function (PSF) automatically calculated from the imaging parameters. Split-GFP complementation signals appear in the region of ER tubules where 19K and IRE1α colocalize. Flag and 19K-positive cells (*n* = 492 for IRE1α-LD and *n* = 40 for PERK-LD) were plotted for GFP intensity, and the percentage GFP-positive cells is indicated. Data are presented as median with first and third quartiles, and whiskers as boxes and lines, respectively. Statistical tests were performed using Wilcoxon two-sided nonparametric test, and two independent experiments gave similar results (lower right panel).

To test if 19K interacted with IRE1α, we stably expressed C2 19K in human embryonic kidney (HEK) 293 cells, yielding a reticular ER-like pattern in the cytoplasm (Supplementary Fig. 4a). Immunoprecipitation of 19K by the monoclonal IgG antibody 3A9 significantly enriched IRE1α compared with pulldowns with a control IgG, suggesting a complex of IRE1α and 19K. We next tested if the lumenal domains of C2-19 K and IRE1α interacted in the ER. Tripartite split-GFP fluorescence complementation assays were used in cells expressing the 19K-LD and IRE1α or PERK-LD. The Flag-IRE1α containing the 20-amino-acid domain GFP10 at the C terminus and the C2 19K-LD with the C-terminal 18 amino acids of GFP11 gave rise to reticular ER-like green fluorescence signals in transfected cells expressing signal sequence containing GFP1–9 with a HDEL ER retention signal (Fig. 4a, b). Green fluorescence signals colocalized with the anti-19K and anti-Flag immunostainings validating the GFP complementation signals. In contrast, no green fluorescence was obtained with Flag-tagged PERK-GFP10-LD, although both 19K and PERK localized in reticular cytoplasmic patterns, akin to IRE1α, indicating selectivity of complementation. The 19K-LD of AdV-D8 (D8 19K-GFP11-LD) gave only faint GFP complementation with SS-GFP1–9-HDEL, but signals with SS-GFP1–10-HDEL as strong as C2 19K-GFP11-HDEL, indicating robust expression of both C2 and D8 19K-GFP11-HDEL fusion proteins (Supplementary Fig. 4b, c). Collectively, the data show that the C2 19K-LD closely and specifically associates with IRE1α, activates IRE1α, and triggers XBP1 splicing.

### XBP1s boosts E1A and 19K, IFN-γ inhibits E1A expression

**Fig. 5:**
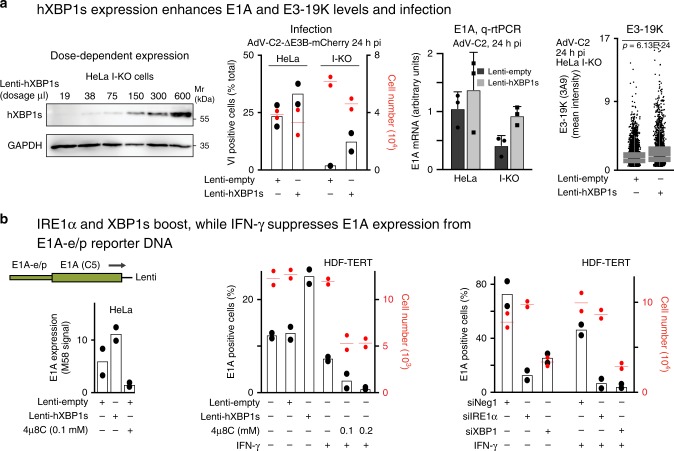
XBP1s enhancement of E1A and 19K levels depends on the E1A promoter. **a** The expression of human XBP1s (hXBP1s) enhances E1A and 19K expression and infection. Dose-dependent expression of hXBP1s in HeLa I-KO (IRE1α-knockout) cells transduced with lentivirus vectors for 72 h (first panel). AdV-C2-dE3b-mCherry infection (MOI 100) is rescued in HeLa I-KO cells by hXBP1s expression, where the cells were transduced with lentiviruses for 2 days and infected with AdV-C2 for 24 h (second panel). Data are presented as mean from two technical replicates, and two independent experiments gave similar results. qrt-PCR showing the rescue of E1A mRNA by hXBP1s overexpression in AdV-C2-(MOI 75) infected HeLa I-KO cells. Data are presented as mean ± SD from three independent experiments (third panel). Increased 19K protein levels by hXBP1s expression in AdV-C2-(MOI 75) infected HeLa I-KO cells (*n* = 3627 and 2317 for lenti-empty and lenti-hXBP1s, respectively, fourth panel). Data show the medians and first and third quartiles, and whiskers as boxes and lines, respectively. Statistical analyses were done by two-tailed Wilcoxon nonparametric tests with significance *p < 0.001. Two independent experiments gave similar results. **b** E1A expression from the E1A-e/p can be increased by ectopic XBP1s, or reduced by the IRE1α inhibitor 4µ8C in HeLa cells (left panel). A similar experiment was carried out in HDF-TERT cells including IFN-γ to suppress the *E1A* promoter/enhancer expressed from a lentivirus vector (middle panel). E1A expression in HDF-TERT cells is reduced by RNA interference against IRE1α and XBP1, but not by nontargeting siRNA (siNeg1, 20 nM siPools, right panel). Error bars represent standard deviations. For all the graphs, data are presented as mean from two technical replicates, and two independent experiments gave similar results.

We next asked if XBP1s directly enhanced AdV infection. Lentivirus-mediated transduction of human XBP1s gave a dose-dependent increase of XBP1s in HeLa I-KO cells, and increased AdV-C2 infection (Fig. 5a). This was confirmed by qrt-PCR measurements of E1A mRNA and 19K protein levels (Fig. 5a). The XBP1s enhancement of E1A was dependent on the activation domain of XBP1s, as demonstrated by expression of mutant murine XBP1s lacking the activation domain due to a premature stop codon after the leucine zipper domain (Supplementary Fig. 5a). Both wild-type and mutant XBP1s mRNAs were expressed similarly, although we could only detect wild-type XBP1s in western blots with an antibody against the C-terminal region containing the transactivation domain. The ectopic expression of XBP1s enhanced the E1A protein levels under the viral E1A-e/p in HeLa and HDF-TERT cells (Fig. 5b). The application of the IRE1 nuclease inhibitor 4µ8C reduced E1A expression, indicating that both XBP1s and IRE1α boost E1A expression in the absence of other AdV gene products (Fig. 5b). In contrast to XBP1s and IRE1α, IFN-γ reduced E1A expression. Notably, the XBP1s-binding sites on the E1A-e/p are separated from the E2F co-repressor-binding sites controlled by IFN-γ^36^. The enhancement of E1A expression by IRE1α and XBP1s was further confirmed by small-interfering RNAs targeting IRE1α and XBP1, which reduced the lentivirus-based E1A expression in both untreated and IFN-γ-treated HeLa or HDF-TERT cells (Fig. 5b). The treatment of HDF-TERT cells with IFN-γ also suppressed AdV-C5 long-term infection, as indicated by strongly reduced expression of the E4-GFP-Orf4 fusion protein (Supplementary Fig. 5c), in agreement with the literature^36^.

Since reduced XBP1s levels may affect cell proliferation^57,58^, we checked if XBP1s levels affected the growth of HDF-TERT cells. Label-free, real-time impedance measurements (xCELLigence) reporting on cell numbers, cell adhesion, and cell–cell interactions^59^ indicated that both 4µ8C and RNAi against XBP1 reduced cell numbers upon 2–3 days of treatment. The washout of 4µ8C 5 days post treatment restored cell proliferation, indicating that 4µ8C affected cell growth but not viability, whereas XBP1s RNAi led to cell death 4–5 days post treatment (Supplementary Fig. 5d). We conclude that IRE1α KO cells exhibit reduced levels of basal XBP1s, and do not increase XBP1s upon ER stress induction. XBP1 on the other hand is essential for cell viability. Ectopic XBP1s enhances E1A expression, even if E1A is expressed in the absence of other viral genes.

### XBP1s binds to the E1A-e/p and promotes E1A expression

**Fig. 6:**
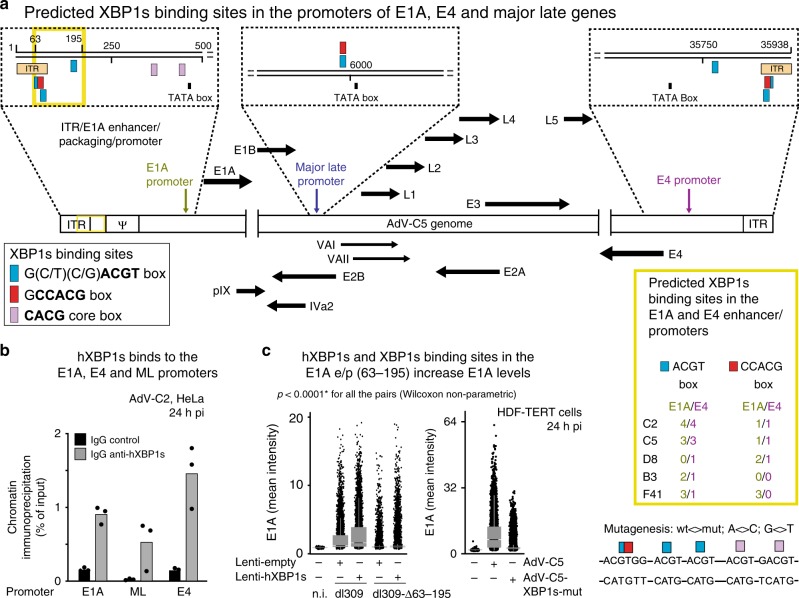
XBP1s binds to predicted sites on the AdV E1A and E4-e/p. **a** Schematics depicting the XBP1-binding sites (blue and red boxes) and consensus-binding sequences on the E1A, E4, and major late (ML) promoters in AdV-C5 genome. The letter psi denotes the packaging sequence near the inverted terminal repeat (ITR). The yellow box on the right shows the conservation of predicted XBP1s-binding sites in the *E1A* and *E4* promoters in different human AdV types. **b** Chromatin immunoprecipitations from AdV-C2-infected cells using anti-XBP1s and control IgG antibodies. The data show the fold enrichment of XBP1s on the AdV-C2 promoters (in particular the E1A and the E4 promoters) with anti-XBP1s versus control antibodies, as calculated using precleared chromatin as input^36^. Data show the means from three technical replicates. Two independent ChIP experiments gave similar results. **c** The deletion and mutagenesis of the XBP1s-binding sites on the E1A enhancer/promoter (e/p), nucleotide positions 63–195 reduce the E1A expression in normal cells and cells ectopically expressing XBP1s. E1A expression analyses in control or hXBP1s-transduced HDF-TERT cells infected with C5-dl309 or the mutant virus C5-dl309-∆63–195, and E1A expression analysis in AdV-C5 and mutant (mut) AdV-C5-XBP1s-mut-infected HDF-TERT cells (MOI 75000). Eight thousand cells per condition, including non-infected cells (n.i.), were chosen by random sampling of the total data. The mutagenized E1A-e/p region in AdV-C5-XBP1s-mut virus is shown on the right side with A<>C and G<>T substitutions. Data show the medians and first and third quartiles, and whiskers as boxes and lines, respectively. Statistics were performed using two-tailed Wilcoxon nonparametric test with *p < 0.0001. Three independent experiments gave similar results.

XBP1s is an integral element of the UPR, and binds to the promoters of genes restoring homeostasis upon ER stress^60,61^. We identified multiple elements of the ACGT and the CCACG-binding box motifs in the *E1A* and the *E4* promoters, and one of each in the major late (ML) promoter, conserved in several AdV species, including B, C, D, and F (Fig. 6a). To test if XBP1s bound to the E1A-, E4-, and ML promoters, ChIP analysis of AdV-C2-infected cells was performed. XBP1s antibodies enriched the *E1A, MLP*, and *E4-*e/p regions fivefold to sevenfold over isotype control antibodies (Fig. 6b). The deletion of the four *E1A* XBP1s-binding sites in *E1A* significantly reduced E1A expression in HDF-TERT cells from dl309_Δ63–95 compared with dl309, most prominently, if XBP1s was overexpressed (Fig. 6c). This result was supported by site-specific mutagenesis of the five XBP1s-binding sites (ACGT, CCACG, and CACG boxes) yielding AdV-C5-XBP1s-mut, which showed strongly attenuated E1A expression compared with AdV-C5 (Fig. 6c). In accordance, the vDNA copy numbers were four logs reduced in dl309_Δ63–195-infected HDF-TERT cells compared with dl309 at 216 hpi (Supplementary Fig. 6a). Similar result was obtained with AdV-C5-XBP1s-mut compared with AdV-C5. The reduced cytopathic effects of dl309_Δ63–195 and AdV-C5-XBP1s-mut compared with dl309 and AdV-C5 were confirmed by impedance measurements (Supplementary Fig. 6b). We conclude that XBP1s binds to the E1A-e/p, transactivates E1A transcription, and drives lytic AdV infection.

### The IRE1α–XBP1s axis enhances persistence

**Fig. 7:**
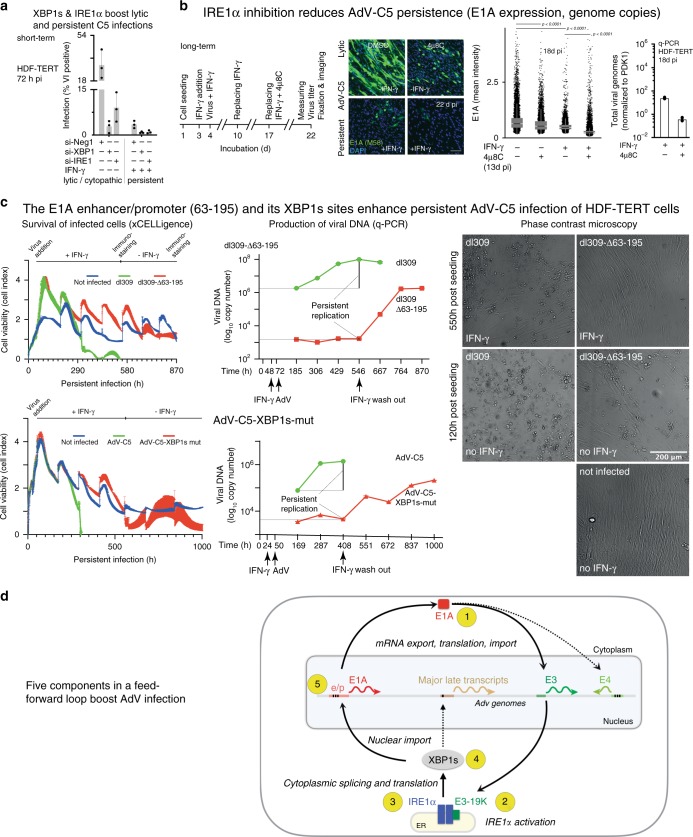
IRE1α and XBP1s facilitate persistent and lytic AdV infections. **a** Reduction in late AdV-C5 protein VI expression (MOI 180) of HDF-TERT cells upon RNA interference against IRE1α and XBP1 in the presence or absence of IFN-γ, including nontargeting siRNA (siNeg1). Data show the means ± SD from three independent experiments (*n* = 3). **b** E1A expression of AdV-C5-infected HDF-TERT cells (MOI 200, 37 °C, 1 h) with or without 500 IU IFN-γ, or IRE1α RNase inhibitor 4µ8C (100 µM) at 13 days pi was analyzed by immunofluorescence showing representative images (left) and a scatterplot with 15,000 cells per condition (middle). Data show the median, first and third quartiles, and whiskers as boxes and lines, respectively. Significance was assessed with two-tailed Wilcoxon nonparametric test (middle panels). Virus titers (q-PCR, right panel) were determined after 5 days of incubation with the drug (18 d pi). Q-PCR data show the means from two technical replicates. Two independent experiments gave similar results. Scale bar, 100 µm. **c** xCELLigence impedance plots showing HDF-TERT cell viability upon dl309 or dl309-63/195 and AdV-C5 or AdV-C5-XBP1s-mut infections (MOI 200). Cells were seeded on xCELLigence E-16 plate, and impedance readout for cell viability was measured live at 15-min intervals. Data show the means ± SD from three technical replicates. Two independent experiments gave similar results (left row). Experimental conditions and virus amount were as in panel **b** Genome copy numbers of virions released to the supernatant from the same experiment. Data show the means from two technical replicates. Two independent experiments gave similar results (middle row). Representative phase-contrast images of parallel samples imaged live for dl309 or dl309-63/195 infections (images on the right side, scale bar, 200 µm). **d** Schematic model depicting AdV infection under the control of a five-component feedforward loop. (1) The immediate early E1A protein transactivates early promoters. including the E3 and E4, giving rise to the 19K glycoprotein (2). Activation of IRE1α by 19 K increases XBP1s mRNA, and XBP1s protein (3), which translocates into the nucleus, and binds to the E1A enhancer/promoter (e/p) of the episomal viral genome (4). Binding of XBP1s to the E1A-e/p increases the E1A levels (5), which enhances output from the E3 promoter, enhances the 19K levels, and maintains a feedforward loop supporting viral persistence and lytic infection.

We next explored if IRE1α–XBP1s controlled persistent AdV infections of HDF-TERT cells. Short-term (72 h) persistent infection in the presence of IFN-γ reduced the expression of protein VI about tenfold, which was further reduced by RNA interference against either IRE1α or XBP1, also in the presence of IFN-γ (Fig. 7a). In long-term infections (22 days), 4µ8C reduced both lytic (without IFN-γ) and persistent (in the presence of IFN-γ) infections, as determined by E1A expression and vDNA copy numbers (Fig. 7b).

To test if persistence depended on the E1A-e/p, we used impedance measurements and q-PCR to monitor infections with dl309_Δ63–195 and AdV-C5-XBP1s-mut lacking the major XBP1s-binding sites in the E1A-e/p due to deletion or point mutations, respectively. The E1A-wild-type viruses dl309 and AdV-C5 were used as controls. HDF-TERT cells were seeded onto xCELLigence plates, treated with IFN-γ, infected 36 h later, and incubated in the presence of IFN-γ until 22.5 days post seeding, followed by IFN-γ washout and incubation for another 13.5 days. Multiplicities of infection (MOI) of both viruses was chosen such that similar cytopathic effects occurred without IFN-γ treatment at 120 h post seeding (Fig. 7c, right panel, see also E1A expression data in Supplementary Fig. 7b). While dl309 and AdV-C5 infections led to cell death after 12.5 and 11 days, respectively, in the presence of intermittent amounts of IFN-γ, the dl309_Δ63–195 and the AdV-C5-XBP1s-mut-infected cells remained viable throughout the course of the experiment to about 36 days (870-h time point), akin to uninfected cells (Fig. 7c). Under IFN-γ, the titers of dl309_Δ63–195 and AdV-C5-XBP1s-mut did not rise (middle panel), and the cells remained viable as shown by phase-contrast microscopy and impedance measurements using xCELLigence, indicative of only low levels of persistence, whereas titers of dl309 or AdV-C5 increased by 10^2^−10^3^-fold, indicating high persistence of *E1A* normal viruses (Fig. 7c). Importantly, upon removal of IFN-γ 22.5 days post seeding, the dl309_Δ63–195 and AdV-C5-XBP1s-mut titers increased several hundred to a thousand-fold 36 days post seeding, while the dl309 or AdV-C5 infections lead to rapid cell death. These results demonstrate that the XBP1s-binding sites in the E1A-e/p support the persistence under IFN-γ. The data show that noncanonical activation of IRE1α maintains a feedforward loop between the ER and the nucleus, gives rise to E1A and 19K expression, maintains AdV persistence, and boosts lytic infection (Fig. 7d).

## Discussion

AdVs encode several immune-modulatory membrane proteins. We showed that the E3-19K glycoprotein of species C2/5 but not of the divergent D8 AdV is necessary and sufficient to selectively activate IRE1α, but not PERK, ATF6, and RIDD, see also ref. ^59^. Activations of PERK and RIDD normally restore homeostasis upon ER stress by phosphorylation of the eukaryotic initiation factor 2 GTPase inhibiting global protein synthesis, and degrading mRNA and microRNAs, respectively^45,62^. This is in accordance with unabated protein production at the time of full IRE1α activation, that is no loss in Flag-hIRE1α, BiP, or β-tubulin, indicating that AdV does not activate the UPR to inhibit protein synthesis. The ensuing 19K expression and association of the 19K-LD with the IRE1α-LD induced IRE1α phosphorylation and XBP1 mRNA splicing yielding XBP1s. These results are compatible with proteomics data showing that XBP1s levels are increased in AdV-infected cells^63^.

Remarkably, AdV-infected cells dissociated BiP from IRE1α before XBP1 splicing, and independent of 19K expression, but dependent on E1A, indicating that the E1A protein, its mRNA, or an E1A-controlled gene product dissociates BiP from IRE1α. This may be akin to a subset of ER-targeted cellular mRNAs, signal recognition particle RNA, ribosomal RNAs, or transfer RNAs, which can directly activate IRE1α^64^. The noncanonical UPR induction by AdV contrasts the known cellular and viral UPR inductions. Lumenal peptides can bind to both BiP and IRE1α, for example, the 8ab protein of SARS-coronavirus triggers the transition of IRE1α from a closed to an open conformation, and leads to IRE1α oligomerization^65^. Consequences of IRE1α activation comprise inflammatory reactions in conjunction with activation of pattern recognition receptors^66^. The LD of C2 19K and IRE1α was in close association based on immunoprecipitations and split-GFP complementation assays. This mode of IRE1α activation contrasts with a recent lipid stress activation model of IRE1α, where BiP binding to IRE1α is not affected^43,67^. Importantly, IRE1α activation by C2 19K was independent of 19K interaction with MHC-I in the ER lumen, indicating that MHC-I sequestration and prevention of CTL-mediated killing of infected cells are not required for IRE1α activation.

The initiation of XBP1 mRNA splicing is a key output from activated IRE1α reviewed in refs. ^9,68^. XBP1s is central in boosting lytic and persistent AdV-C2/5 infections. Lytic infection could be rescued by overexpression of XBP1s in IRE1α KO cells. More specifically, XBP1s was found in a complex with the E1A-e/p in infected cells, and boosted E1A (and also 19K) expression, and lytic infection. The XBP1s-binding sites on the E1A-e/p are distantly located from the E2F-Rb repressor-binding sites^36^, a configuration that allows for coregulation by repressors and stimulators, which we show here is crucial in AdV persistently infected cells in the presence of IFN-γ. The concept of coregulation by activators and repressors is found with herpes viruses and HIV, where transcriptional coregulation occurs during persistence^69,70^. In accordance, the IRE1α nuclease inhibitor 4µ8C, which reduces XBP1s, reduced AdV-C5 persistence in HDF-TERT cells under IFN-γ.

The C2/5 19K glycoprotein of the E3 region together with the transcriptional activator E1A is important to maintain the levels of XBP1s, as indicated by infection of HDF-TERT cells with AdV-C5-Δ19K. 19K activates IRE1α, which increases XBP1s, and XBP1s enhances E1A transcription. This supports both lytic and persistent infection of HDF-TERT cells, as indicated by AdV-C5 mutants lacking functional XBP1s-binding sites in the E1A-e/p. In nonlymphoid cells, the *E3* promoter, which lacks XBP1s-binding sites is controlled by E1A, and in lymphoid cells, it is E1A-independent but NF-κB dependent^71^. This may lead to cell death. For example, TNFα exposure induces ER stress, IRE1α, and NF-κB activation, and exacerbates death signaling through the TNF receptor 1, involving c-JUN N-terminal kinase^72^. If and how AdV antagonizes this signaling pathway is unknown. Alternatively, other host transcription factors could be involved in E3 regulation. For example, SP1, which is induced by XBP1s^60^ has 29 predicted binding sites in the E3 promoter, and could enhance E3 transcription. Regardless of the nature of the E3 promoter regulation, the deletion of the 7.1K and 19K region in AdV-C5 reduced viral persistence in Syrian hamsters^73^. Our data highlight the therapeutic potential of the IRE1α branch of the UPR, and converge on the notion that in Zika virus-infected human and mouse embryos, the UPR was observed in the cerebral cortex of postmortem fetuses, and the IRE1α nuclease inhibitor 4µ8C reduced the microcephaly frequency^74^. In conclusion, the selective activation of IRE1α by 19K provides an environment conducive for AdV persistence, and supports the lytic cycle under conditions of impaired immunity.

## Methods

### Cells and viruses

A549 and HeLa-ATCC cells were obtained from American Type Cell Culture (ATCC). HCE cells were obtained from Dr Niklas Arnberg (UMEA University, Sweden). Human diploid fibroblasts immortalized with telomerase (HDF-TERT)^75^ and other cells were grown at 37 °C in 5% CO_2_ environment in DMEM (Sigma) supplemented with 10% fetal calf serum (FCS). For generation of the AdV-C2_dE3B-mCherry virus, the GFP ORF in AdV-C2_dE3B_GFP^55^ was replaced with the mCherry ORF. The virus genome was cloned into pKSB2^76^, followed by two homologous recombination steps according to the recombineering protocol numbers 1 and 3^77^. Note that the MOI varied between different experiments and viruses depending on the susceptibility of the cells, the nature of the experiment (persistence/lytic/early/late infection readout), and the infection protocol (warm or cold-synchronized infection). For generation of AdV-C5-E3-Δ19K and AdV-C5-XBP1s-mut viruses, AdV-C5 (wt300) DNA^78^ was inserted into pKSB2^76^ followed by two homologous recombination steps according to the recombineering protocol numbers 1 and 3^77^. Insertion of the sequence 5′-ATTTATTGTCAGCTTTTTAAACGCTGGGGTCGCCACCCAAGATGATTTACTAAGTTACAAAGCTAATGTCACCACTAACTGCTTTACTCG-3′ in the second step introduced a complete deletion of the 19K ORF except the first 4 nt, allowing to keep the overlapping stop of the upstream 6.7K CR1a gene (AdV-C5-dE3-19K). Similarly, insertion of 5′-CCTTAATTAAGGGCGGCCGCATTTAAATTAATTAACATCATCAATAATATACCTTATTTTGGATTGAAGCCAATATGATAATGAGGGGGTGGAGTTTGTGCATGTTCGCGGGGCGTGGGAACGGGGCGGGTGCATGAGTAGTGTGGCGGAAGTGTGATGTTGCAAGTGTGGCGGAACACATGTAAGCGACGGATGTGGCAAAAGTGCATGTTTTGGTGTGCGCCGGTGTACACAGGAAGTGACAATTTTCGCGCGGTTTTAGGCGGATGTTGTAGTAAATTTGGGCGTAACCGAGTAAGATTTGGCCATTTTCGCGGGAAAACTGAATAAGAGGAAGTGAAATCTGAATAATTTTGTGTTACTCATAGCGCGTAATATTTGTCTAGGGCCGCGGGGACTTTGACCGTTTCATGGGAGACTCGCCCAGGTGTTTTTCTCAGGTGTTTTCCGCGTTCCGGGTCAAAGTTGGCGTTTTATTATTATAGTCAGCTTCATGGTAGTGTATTTATACCCGGTGAGTTCCTCAAGAGGCCACTCTTGAGTGCCAGCGAGTAGAGTTTTCTCCTCCGAGCCGCTCCGACACCGGGACTGAAAATGAGACATATTATCTGCCACGGAGGTGTTATTACCGAAGAAAT-3′ in the E1A-e/p region introduced XBP1s-binding sites mutagenized using A<>C; G<>T substitution rule (AdV-C5-XBP1s-mut, Fig. 6c). All the indicated MOI were calculated as the estimated number of viruses per cell where virus titer was determined by q-PCR based described in the section “Virus titer estimation.” For specific reagents, see Supplementary Table 1.

### Cas9 knockout of IRE1α in HeLa-ATCC cells

HeLa-ATCC and 293T cells were transduced with Lentiviral vector expressing Cas9 (Addgene, 49535) and a guide RNA against exon 2 of mammalian ERN1 (IRE1α, chromosome 11)^79^. A polyclonal population of both cell lines expressing Cas9 was selected with puromycin (2 µg/ml), and single clones were isolated from HeLa-ATCC. For selection of clonal population, a polyclonal population was seeded at high dilution in a 15-cm dish and allowed to grow into several sparse clones. Using circular rings, small populations from single clones were trypsinized, collected, and seeded on 96-well clear-bottom plates for expansion. After several weeks, DNA from the growing clonal population was extracted, and using primers flanking the target site of Cas9 on exon 2 of IRE1α, PCR was performed, and fragments were ligated into plasmid vector (Bluescript). Positive clones were sequenced, aligned to the wild-type mammalian IRE1α locus (NCBI identifier NC_000017.11 region 64039142–64132469), and the resulting mutations were identified. As IRE1α is triploid in HeLa cells^80^, sequencing of the clone used in this study revealed three distinct mutations, namely insertion of A, frameshift deletion of 23 nt, and in-frame deletion of 15 nt (Supplementary Fig. 1b).

### Lentivirus production, cloning, and transduction

The cDNA inserts for human IRE1α^43^, human XBP1s (Addgene 63680, NCBI Gene ID 7494), mouse XBP1s (NCBI Gene ID 22433), AdV-C5 E1A enhancer, promoter, and E1A expression cassette (1-1701 genomic region from AdV-C5 genome NCBI AC_000008.1) were cloned into PLVX-IRES-Puro (Clontech 632183). Lentiviral vectors were produced in HEK293T cells by transfecting lentiviral construct, pCMVR8.91-Gag-Pol and pVSV-G (Clontech)^81^. Relative titers of different lentiviruses were determined using dose–response of puromycin 72 h post transduction. For transduction of cells, predetermined amounts of lentivectors that gave good expression of the target gene were added to cells at the time of seeding, and transduction was performed for 48 h. Cells were either collected for analyses of the transgene expression levels by western blotting or for chromatin immunoprecipitation or infection assays, as indicated.

For the ectopic expression of AdV-C2 and D8 E3-19K (Genbank AB448767.1) in the absence of other viral factors, an expression system was developed where codon-optimized AdV-C2 E3-19K construct after the N-terminal calreticulin signal sequence (Css) was synthesized by GeneArt/Life Technologies and inserted into pSG5 expression vector^82^ (pSG5-19K-CO Css). For insertion of AdV-D8 E3-19K in the same construct, D8 E3-19K ORF after the wild-type signal sequence was amplified by PCR from purified vDNA and substituted with the C2 E3-19K sequence in pSG5-19K-CO Css plasmid after the Css sequence using NEBuilder^®^ HiFi DNA assembly cloning kit (New England Biolabs). Lumenal domain (LD) 19K-LD-Flag-HDEL mutants of C2 and D8 19K were made by the deletion of the transmembrane and cytoplasmic domains of E3-19K and insertion of Flag-HDEL domain by site-directed mutagenesis using the Q5^®^ Site-Directed Mutagenesis Kit (New England Biolabs). The correctness of all the constructs was verified by sequencing.

### Transfection and infection

For knockdown experiments, siPools^TM^ oligos (siTools Biotech GmbH, Martinsried, Germany) were used. siPools have minimal off-target effects due to subthreshold concentrations of individual dsRNAs^83^. siRNAs were mixed with 9.8 µl of Opti-MEM medium (Invitrogen) and Lipofectamine RNAiMax reagent (0.2 µl/well, Invitrogen), and incubated at room temperature for 5 min in a 96-well plate (Greiner). Ten thousand HeLa-ATCC or HDF-TERT cells were added in 90 µl per well of DMEM medium supplemented with 10% FCS of the 96-well plate and incubated at 37 °C in a 5% CO_2_ environment for 48 h. After this, continuous infection with AdV-C5 or AdV-C2-dE3B_mCherry was carried out for 24, 48, or 72 h. Multiplicity of infection (MOI) was calculated from the absorbance of purified particles at 260 nm in the case of AdV-C5_dl309 or dl309_∆63–195, by the infectious titer determined in A549 cells, or as indicated.

For HeLa cells, the final concentration of 10 nM, and for HDF-TERT cells, 20 nM of siPools were transfected, based on knockdown efficiency. For the rescue of infection in knockout cells, cells were transduced with lentivectors expressing human XBP1s or IRE1α for 48 h, incubated with indicated viruses for 24 h followed by fixation and staining with protein VI or E1A (M58). Control cells were transduced with lentivectors devoid of the transducing gene. Further analysis was done with high-throughput microscopy, western blotting, or quantitative PCR as described below.

For transfection of split-GFP constructs in HEK293T cells, 100 ng of each construct per well of the 96-well plate was mixed with 9.8 µl of Opti-MEM medium (Invitrogen) and Lipofectamine 2000 reagent (0.2 µl/well, Invitrogen), and incubated at room temperature for 5 min after directly spotting on a 96-well plate (Greiner). Ten thousand HEK293T cells were added in 90 µl of DMEM medium supplemented with 10% FCS per well of the 96-well plate and incubated at 37 °C at 5% CO_2_ for 48 h. Cells were fixed 48 h post transfection and stained with anti-E3-19K (3A9) and anti-Flag antibodies followed by imaging and analysis as described in the section called high-throughput imaging and measurement of infection. Representative images of split-GFP constructs for Fig. 4b were taken with a confocal SP5 microscope (Leica) and maximal projection images are shown.

For Neon transfection (Thermo Fisher Scientific) of E3-19K constructs in HeLa-ATCC cells, 5 µg of the plasmid DNA was mixed with Resuspension buffer (Neon) at a density of 0.5 × 10^7^ cells/ml and filled in the Neon Tip (100 µl of plasmid plus buffer) attached to the Neon Pipette. Neon Tube was filled with electrolytic buffer and attached to the Neon Pipette Station. The cells were electroporated at 1005-V pulse voltage, at 35-ms pulse width, and two pulses. Following this, the cells were resuspended in antibiotics-free DMEM medium supplemented with serum (FCS) and seeded on the 6-well plate for RNA extraction or western blotting or 96-well plate for immunofluorescence staining. Twenty-four hours after transfection, cells were either collected for RNA extraction or fixed and DAPI stained for the calculation of the percentage E3-19K-expressing cells.

### XBP1-splicing assay

Total RNA from HeLa-ATCC and HDF-TERT cells was lysed in Trizol Reagent (Invitrogen) and extracted using the Direct-zol RNA MicroPrep kit (Zymo Research). The cDNA synthesis was carried out with MMLV RT (Promega, M170) using Oligo dT 15 mer primers (Promega, C1101). PCR amplification for XBP1 gene was performed, and products were digested with PstI and treated with T7 Endonuclease (NEB, E3321) to get rid of the hybrid XBP1s–XBP1u bands (Supplementary Fig. 1f). The DNA bands were resolved in 2% agarose gel and images were taken with GeneSnap (Syngene).

### IRE1α and PERK mobility shift assay

Activation of PERK signaling arm of UPR was checked using the electrophoretic mobility shift assay. Hyperphosphorylated forms of PERK were detected by sufficiently resolving the 1-h DTT-(10 mM) treated cell lysates in 4–20% gradient gels (Biorad), and detecting the protein using anti-PERK antibody (Cell Signaling). For separating the phosphorylated from hypophosphorylated forms of IRE1α, 6% polyacrylamide gels containing 25 µM Phos-tag (Fujifilm WAKO) and ZnCl_2_ was used. All buffers were prepared as described by the manufacturer (https://www.igz.ch/downloads/16079/phos-tagtm_sds-page_guidebook_11.pdf), and Phos-tag-bound proteins resolved on the polyacrylamide gels were incubated with 10 mM EDTA for 10 min prior to transferring them to PVDF membranes to reduce the adverse effects of Zn^2+^ on protein transfer efficiency.

### Immunoprecipitation and immunoblotting

HeLa-ATCC, Flag-IRE1α-MEFs, or 293T cells were seeded on 10-cm dishes. Twenty-four hours later, cells were infected with AdV-C2 (MOI 100 for HeLa-ATCC or twofold higher for Flag-IRE1α-MEFs) at 37 °C, 5% CO_2_ for 24 h. Following this, the medium was aspirated and washed with ice-cold PBS several times. Cells were scraped in 600 µl of DMEM and collected in 1.5-ml tubes. Cells were pelleted and resuspended in 600 µl of ice-cold IP lysis buffer (Tris-HCl, pH 8.5, NaCl 150 mM, MgCl_2_ 1 mM, EDTA 1 mM, Nonidet P-40 0.5%, and protease inhibitor cocktail) under agitation at 4 °C for 20 min. The cells were centrifuged at 16,000 × *g* at 4 °C for 10 min, and the supernatant was collected. Fifty microliters of the cell lysates were kept for an input control sample and the rest was precleared with protein-A sepharose beads (Abcam, ab193256). Precleared lysates were incubated with anti-3A9^84^, anti-Flag (Sigma, F7425), or an equivalent amount of IgG control overnight at 4 °C. The next day, 30 µl of Protein-A sepharose beads were added per sample and incubated at 4 °C for 1 h with agitation. Beads were spun down, the supernatant removed, and beads washed in IP lysis buffer several times. After this, the beads were mixed with 2× SDS lysis buffer (10% w/v), boiled at 95 °C for 5 min, and the supernatant was collected. Samples were resolved with polyacrylamide gel electrophoresis after addition of DTT (50 mM) for anti-Flag and without DTT for anti-3A9 immunoblotting. For immunoprecipitations of Flag-IRE1α from Flag-IRE1α-expressing MEFs and lumenal domain of IRE1α-expressing HeLa cells, the ratio of BiP to IRE1α in the IP fraction was normalized to their respective levels in the lysate.

### Tripartite split-GFP protein interaction assay

The tripartite split-GFP complementation assay was modified based on earlier protocols^85,86^. For the construction of tripartite split-GFP plasmids, lumenal domains of E3-19K from AdV-C2 or D8, and Flag-tagged human IRE1α or PERK was PCR amplified (Supplementary Table 2) and inserted into pcDNA 3.1 plasmid having the GFP11 and 10 domains, respectively. The rest of the GFP1–9 and GFP1–10 domain was inserted after Css at the N terminus and ER retention signal HDEL at the C terminus and cloned in pcDNA 3.1. For full-length split-GFP constructs shown in Supplementary Fig. 4b, IRE1α-GFP11 and 19K-GFP10 pair was chosen due to the better signal:noise ratio of this pair. For IRE1α, GFP11 domain was inserted at the linker region after the transmembrane domain of IRE1α. The rest of the GFP domains 1–9 were cloned in pcDNA 3.1. One hundred nanograms of all the plasmids were transfected in HeLa or 293T cells using Lipofectamine transfection method. Forty-eight hours later, cells were fixed with 3% paraformaldehyde (PFA) and stained with anti-Flag and anti-E3-19K (3A9) antibodies and imaged with high-throughput and confocal microscopes to measure the expression levels. The number of cells was determined with DAPI staining. Total tripartite split-GFP puncta were identified using custom-made CellProfiler script (Supplementary Table 4) and plotted using JMP 13.

### Chromatin immunoprecipitation

HeLa-ATCC cells (~1 × 10^6^ cells) grown on a 10-cm dish were transduced with lentivirus vector expression human XBP1s. Forty-eight hours after transduction, cells were infected with AdV-C5 at a MOI of 1200 at 37 °C for 1 h. Unbound virus was washed away, and infection was continued for 23 h after which the medium was replaced with 5 ml of DMEM, and cross-linking with 333 µl of 16% PFA was done for 10 min and 37 °C. Cross-linking was stopped by the addition of 761.85 µl of glycine (125 mM) at RT for 5 min. After this, cells were washed twice with ice-cold PBS, scraped off the plate, and collected with 600 µl of PBS. Cells were pelleted by centrifugation at 1000 *g* at 4 °C for 5 min and resuspended in 600 µl of SDS lysis buffer (50 mM Tris-HCl, pH 8.0, 10 mM EDTA, 1% SDS, and freshly mixed with protease inhibitor cocktail, Roche 4693159001) at 4 °C for 10 min. Shearing of the cells was performed with Covaris S2 using Duty cycle 10, intensity 5, cycles 3, cycles/burst 200, and time/cycle of 60 s. Samples were centrifuged at high speed to remove cellular debris. One microgram of chromatin was kept for input determination. Ten micrograms of chromatin-containing cell lysates were diluted tenfold with dilution buffer (Tris-HCl, pH 8.0, NaCl 167 mM, EDTA 1.2 mM, SDS 0.01%, and Triton X-100 1.1%) and precleared with protein-A agarose/salmon sperm DNA slurry (Millipore) with rotation at 4 °C for 1 h. Cell lysates were spun to remove precleared complexes and incubated with 3 µg of anti-XBP1s (BD Biosciences) and Rabbit-IgG isotype control (Thermo Fisher Scientific) overnight at 4 °C with rotation. Immune complexes were captured by incubation with protein-A agarose/salmon sperm DNA slurry (Millipore) at 4 °C for 2 h and pelleted by centrifugation (2000 × *g* for 1 min). Washing with low-salt buffer (20 mM Tris-HCl, pH 8.0, NaCl 167 mM, EDTA 150 mM, SDS 0.1%, and Triton X-100 1%), high-salt buffer (20 mM Tris-HCl, pH 8.0, NaCl 500 mM, EDTA 2 mM, SDS 0.1%, and Triton X-100 1%), LiCl wash buffer (10 mM Tris-HCl, pH 8.0, LiCl 0.25 M, EDTA 1 mM, SDS 1%, Triton X-100 0.5%, and sodium deoxycholate 1%), and TE buffer (Tris-HCl, pH 8.0, 10 mM EDTA, 1 mM) was performed, and elution with elution buffer (NaHCO_3_ 100 mM, SDS 1%) was done by incubation with rotation at room temperature for 15 min. Reverse cross-linking for formaldehyde complexes was done by incubation with 5 M NaCl, 0.5 M EDTA, 1 M Tris-HCl, and proteinase K (20 mg/ml) at 65 °C overnight. DNA was extracted using the Qiagen blood and tissue extraction kit and subjected to quantitative PCR using primers listed in Supplementary Table 2. Data were plotted as fold enrichment relative to input.

### Virus titer estimation

Long-term persistent AdV-C5-infected HDF-TERT cells were washed with PBS and collected after scraping of the cells from the plate. DNA extraction was performed with Qiagen blood and tissue kit (Qiagen, #69504), and viral genome copy numbers were determined using quantitative PCR with primers on the AdV *E1A* enhancer region (Fwd 5′-GGTGGAGTTTGTGACGTGG-3′ and Rev 5′-CGCGCGAAAATTGTCACTTC-3′). First the relative abundance of *E1A* templates was calculated using ∆∆Ct method and normalized to the cellular PDK1 gene (see Supplementary Table 2). Afterward, absolute viral gene copy numbers of one of the infected samples were calculated with an *E1A* standard curve using a plasmid DNA containing the *E1A* promoter and enhancer region followed by relating it to the relative fold difference derived from the ∆∆Ct method above. Details of the quantitative PCR are mentioned in the section about quantitative PCR.

### Establishment of persistent AdV infection

HDF-TERT cells were incubated with IFN-γ (500 IU/ml) for 24 h prior to infection. Infection with AdV-C5 (MOI 25) was performed in the presence of IFN-γ for 14 h; cells were washed and incubated with IFN-γ. For establishment of long-term persistence, the medium and fresh IFN-γ were replaced every 5 days until the end of the experiment^36^.

### Promoter activity assays

The AdV-C5 *E1A* gene under the E1A enhancer and promoter elements (genomic region 1-1701 of AC_000008.1) were cloned in pLVX-IRES-Puro lentivector (Clonetech) and used to transduce HeLa-ATCC and HDF-TERT cells along with empty and human XBP1s lentivectors. Forty-eight hours post transduction, 4µ8C (100 µM) or IFN-γ (500 IU) were added to the cells. Seventy-two hours post transduction, cells were fixed with PFA and stained with anti-E1A (M58). The percentage of E1A-expressing cells was determined using custom-made CellProfiler script and data analyzed with Knime software.

### High-throughput imaging and measurement of infection

After fixation and staining, cells were imaged with high-throughput wide-field microscope (Molecular Devices IXM-XL). DAPI staining was used to segment the nuclear mask for the cells, and the intensity of E1A (M58 and 73) and protein VI was measured over this mask using CellProfiler^87^. For the reticular staining of split-GFP and E3-19K staining, nuclear mask was expanded by ten pixels, and E3-19K intensity was measured over this ring-shaped object. The separation of mCherry, E1A, protein VI, or E3-19K-expressing and nonexpressing cells was calculated with KNIME, and graphs were plotted either in JMP version 13 or GraphPad Prism version 8.

### Western blotting

At the time of anlaysis, cell lysates were collected in lysis buffer (0.2 ml of 200 mM Tris, pH 8.8, 20% glycerol, 5 mM EDTA, 50 mM DTT, 5% SDS, and 0.02% bromophenol blue) by boiling at 95 °C and shearing through G21 needles (Sterican). Proteins were resolved in polyacrylamide gels and transferred to a polyvinyldifluoride membrane (Amersham). Blocking of unspecific protein-binding sites of the membrane was done with 5% milk powder. Primary antibody incubation was performed at 4 °C, followed by horseradish peroxidase (HRP)-tagged secondary antibody incubation at room temperature. Protein bands were visualized by incubation with chemiluminescent reagent (Amersham) and the Amersham Imager 600.

### Quantitative rt-PCR

Cells were lysed in Trizol Reagent (Invitrogen) and total RNA extracted using the direct-zol RNA MicroPrep kit (Zymo Research). cDNA synthesis was carried out with MMLV RT enzyme (Promega, M170) using Oligo dT 15 mer primers (Promega, C1101). The amplification was done using SYBR Green JumpStart Taq ReadyMix in Applied Biosystems Quant Studio 3 Real-Time PCR System with primers listed in Supplementary Table 2. For ChiP experiments, the relative quantification procedure of the Pfaffl method was used to convert the average Ct values for each sample to relative fold-change information^88^.

### xCELLigence impedance measurements

For measuring cell toxicity of AdV and chemicals, the xCELLigence impedance measurement (Roche Applied Science and ACEA Biosciences) was used^59,52^. For AdV toxicity in lytic or persistent infections, cells were seeded on E-16 plates for 48 h, followed by incubation with IFN-γ (persistent infection) for 24 h and incubation with AdV at 37 °C for 1 h. Cells were either further incubated with IFN-γ (persistent infection), or with normal medium (lytic infection), and cell index measurements were recorded live at intervals of 15 min. For assessing the effects of 4µ8C on cell viability, cells were seeded and incubated with 4µ8C for 7 days followed by drug washout.

### Click chemistry and visualization of single viral DNA dots

HeLa wild-type and I-KO cells were seeded on alcian-blue-coated coverslips and infected with EdC-labeled AdV-C5 virus at 37 °C for 1 h, followed by washout of unbound virus and further incubation at 37 °C for 1.5 h. Cells were fixed with formaldehyde and analyzed for capsid-free vDNA by copper-catalyzed click chemistry^39^. Viral capsids were stained with antihexon antibody (9C12) and samples imaged with an SP8 confocal microscope (Leica)^52^. Maximal projections of signals were analyzed with CellProfiler where DAPI staining was used to identify a nuclear mask together with overexposed hexon signals to segment the cell boundaries. Viral capsids, vDNA over the nucleus and in the cytoplasmic area was counted, and the distribution of capsid-free vDNA over nuclei and the cytoplasm calculated.

### Prediction of SP1-binding sites in the E3 promoter

An overall consensus around the GC-box core element GGGCGG of SP1-binding sites can be predicted at http://tfbind.hgc.jp/^89^. Accordingly, up to 34 putative binding sites around the GC box were predicted in the E3 promoter, and 29 of them have a predicted consensus match for SP1 binding.

### Quantifications and statistical analysis

Statistical analyses were performed using JMP version 13. For statistical analysis used in Figs. 5a and 6c, pairwise Wilcoxon nonparametric tests were performed.

We declare that no data have been excluded from experimental replicates. Random sampling in Figs. 6c and 7b as well as S4 d was done to keep an even number of cells analyzed between samples.

### Reporting summary

Further information on research design is available in the Nature Research Reporting Summary linked to this article.

## Supplementary information

Supplementary Information

Peer Review File

Reporting Summary

## Data Availability

The data supporting the findings of this study are available within the article and its Supplementary Information files, or are available from the authors upon request. The source data underlying Figs. 1–3 and 5a, and Supplementary Figs. 1e, 2b–e, 3a, b, d–i, 4a, and 5a are provided as Source Data files.

## Source Data

Source Data

## Code availability

Lists of the raw data used in the figures and the codes/scripts used to analyze them are deposited at Mendeley Data. The details are provided in Supplementary Tables 3 and 4.
