# Supplementary material for: The UPR sensor IRE1α and the adenovirus E3-19K glycoprotein sustain persistent and lytic infections
Source: Nat Commun. 2020 Apr 24;11:1997. doi: 10.1038/s41467-020-15844-2 (PMC7181865; doi:10.1038/s41467-020-15844-2)
Supplement: Supplementary file 1 — Supplementary Information [file 41467_2020_15844_MOESM1_ESM.pdf]

Supplementary Information

The UPR sensor IRE1α and the adenovirus E3-19K glycoprotein sustain persistent and lytic infections

Prasad et al.

Supplementary figures and legends

Supplementary Figure 1: Construction and characterization of the HeLa I-KO cells, and depiction of the IRE1α activation assay measuring XBP1 splicing. (Relates to Figure 1).

a. Schematics showing the genomic locus targeted by the guide RNA for the construction of IRE1α knock-out HeLa cells (HeLa I-KO) by CRISPR-Cas9.

b. Mutations in the monoclonal HeLa I-KO cells introduced by CRISPR/Cas9 to the three alleles of IRE1α in HeLa cells. An insertion of an adenosine (A), and a 23-nucleotide (nt) deletion, respectively, resulted in out-of-frame modifications, whereas a 15-nt deletion caused an in-frame deletion.

c. Cell-associated AdV-C5 and nuclear import of incoming viral DNA are not affected in HeLa I-KO cells (n=15 for HeLa and n=12 for HeLa I-KO). Nuclear vDNA was scored as hexon-free vDNA puncta over the DAPI mask. Centre line of the box plot indicates median with 1st and 3rd quartiles and whiskers shown as boxes and lines, respectively. Significance was assessed with two-tailed Wilcoxon non-parametric test. Scale bar, 10 μm.

d. XBP1 splicing assay and the formula to determine the percentage of spliced XBP1 {Han, 2009, 19665977}. The T7 endonuclease removes the hybrid XBP-1u-1s band (*) above the 1s band (two panels on the right). Four independent experiments gave similar results.

e. XBP1 splicing is enhanced in AdV-C2 and C5 infection of HeLa cells (MOI 75). * denotes a background product. Three independent experiments gave similar results. Source data are provided as a source data file.

f. Splicing of XBP1s induced by Golgicide A (GCA) requires IRE1α. HeLa I-KO cells show lesser XBP1 splicing than wild type HeLa cells upon 5 h incubation with GCA (20 μM). Three independent experiments gave similar results.

g. Induction of XPB1s occurs in AdV-C5 infected WI38 cells (MOI 75). Three independent experiments gave similar results.

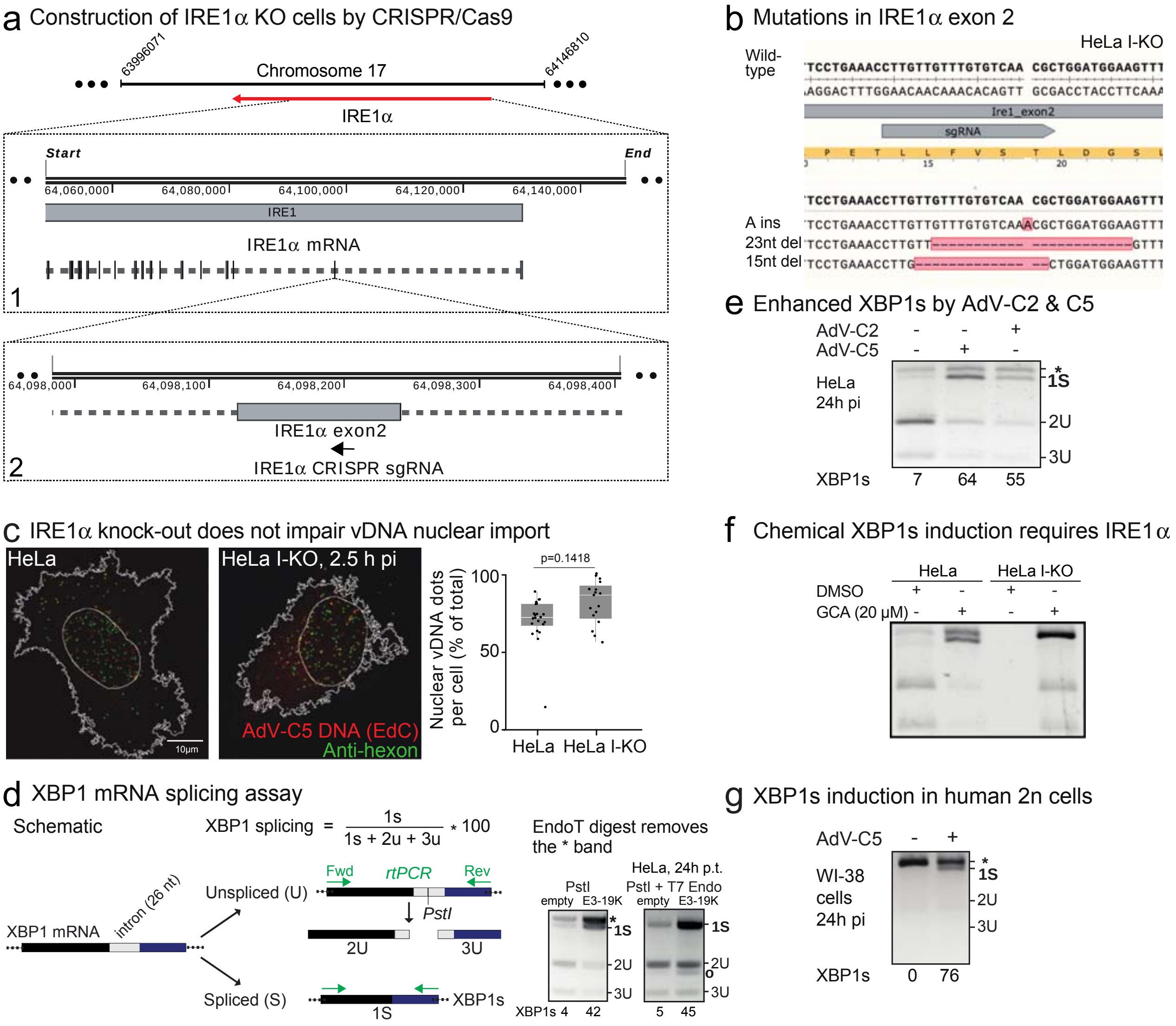
a Construction of IRE1α KO cells by CRISPR/Cas9
63996071
64146810
Chromosome 17
IRE1α
Start
End
64,060,000
64,080,000
64,100,000
64,120,000
64,140,000
IRE1
IRE1α mRNA
1
64,098,000
64,098,100
64,098,200
64,098,300
64,098,400
IRE1α exon2
IRE1α CRISPR sgRNA
2
b Mutations in IRE1α exon 2
HeLa I-KO
Wild-type
Ire1_exon2
sgRNA
A ins
23nt del
15nt del
c IRE1α knock-out does not impair vDNA nuclear import
HeLa
HeLa I-KO, 2.5 h pi
10µm
AdV-C5 DNA (EdC)
Anti-hexon
p=0.1418
Nuclear vDNA dots per cell (% of total)
100
50
0
HeLa
HeLa I-KO
d XBP1 mRNA splicing assay
Schematic
XBP1 splicing = 1s / (1s + 2u + 3u) * 100
EndoT digest removes the * band
XBP1 mRNA
intron (26 nt)
Unspliced (U)
Spliced (S)
Fwd
rtPCR
Rev
PstI
2U
3U
1S
XBP1s
HeLa, 24h p.t.
PstI
PstI + T7 Endo
empty
E3-19K
XBP1s 4
42
5
45
e Enhanced XBP1s by AdV-C2 & C5
AdV-C2
AdV-C5
HeLa 24h pi
XBP1s
7
64
55
f Chemical XBP1s induction requires IRE1α
HeLa
HeLa I-KO
DMSO
GCA (20 µM)
g XBP1s induction in human 2n cells
AdV-C5
WI-38 cells 24h pi
XBP1s
0
76

Supplementary Figure 2: Displacement of BiP/Grp78 from IRE1α by AdV, and attenuation of IRE1α activation by ectopic expression of BiP/Grp78 in canonical UPR. (Relates to Figure 2d, e).

a. AdV infection does not induce RIDD. HeLa cells were either treated with DTT (2 and 10 mM) for 4 h or infected with AdV-C5 for 24 and 48 h. Total RNA was extracted and XBP1s and human Bloc1S1 mRNA levels were measured relative to GAPDH and TBP. Data present the means +/- SD from three independent experiments. Source data are provided as a source data file.

b. BiP-IRE1α dissociation and XBP1s correlate in conventional UPR. Co-immunoprecipitation of Flag-IRE1α and BiP in IRE1α-KO MEFs expressing Flag-IRE1α upon treatment with DTT (10mM) for 1, 2 and 4h and immunoblotting with anti-IRE1α, anti-BiP/Grp78 and anti-β-tubulin antibodies. Aliquots of the corresponding cells were also analysed for XBP1 splicing, and results expressed as % XBP1s. Two independent experiments gave similar results. Source data are provided as a source data file.

c. BiP-IRE1α dissociation in AdV infection of HeLa cells. Lentivirus expressing full-length Flag-IRE1α was transduced in HeLa I-KO cells for 48 h, followed by DTT treatment for 1 h, or AdV-C5 infection for 4 h (MOI 200). Co-immunoprecipitation of BiP-Flag-IRE1α followed by immunoblots was performed as described in (a). The results show that ectopic overexpression of IRE1α increases XBP1 splicing. AdV infection dissociates BiP from IRE1α without increasing XBP1 splicing, while DTT treatment dissociates BiP from IRE1α and increases XBP1 splicing. ** denotes a background product. Two independent experiments gave similar results. Source data are provided as a source data file.

d. Time-course of XBP1 splicing induction in HeLa cells, infected with AdV-C5 (MOI 200) for 4, 8 and 16 h. Three independent experiments gave similar results. Source data are provided as a source data file.

e. Ectopic expression of BiP/Grp78 does not reduce XBP1 splicing in AdV infection. Control and HeLa cells transfected with BiP/Grp78 were infected with AdV-C2 for 24 and 48 h, and analyzed by Western blotting for BiP expression relative to GAPDH, as well as XBP1 splicing was analysed in the corresponding samples. Three independent experiments gave similar results. Source data are provided as a source data file.

f. Ectopic expression of BiP/Grp78 attenuates IRE1α activation in conventional UPR. Control and BiP overexpressing HeLa cells were treated with Tg (10 μM) for indicated times, and XBP1 splicing was analysed. Two independent experiments gave similar results.

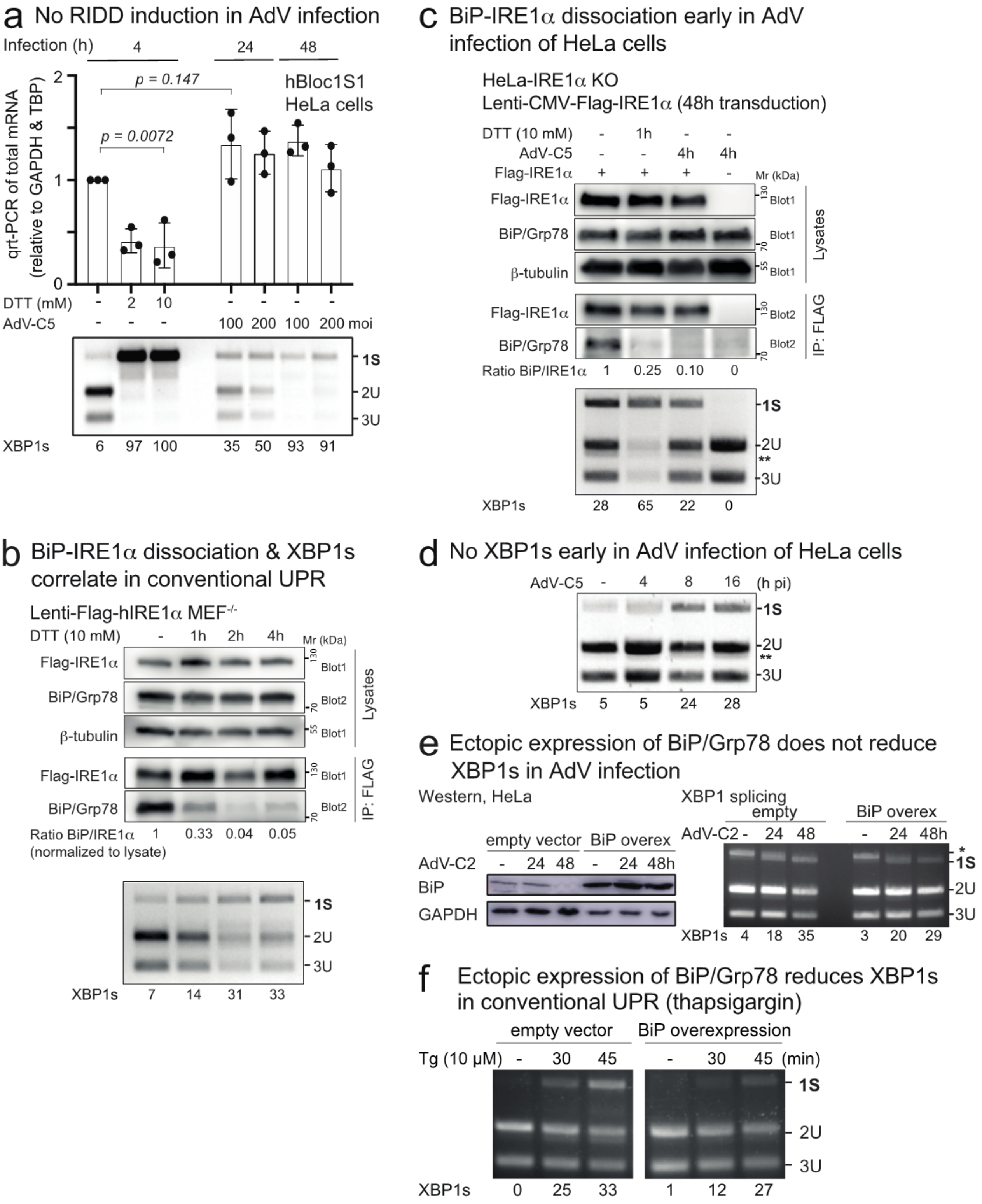
a No RIDD induction in AdV infection
Infection (h) 4 24 48
p = 0.147
p = 0.0072
hBloc1S1
HeLa cells
qrt-PCR of total mRNA
(relative to GAPDH & TBP)
DTT (mM) - 2 10 - - - -
AdV-C5 - - - 100 200 100 200 moi
1S
2U
3U
XBP1s 6 97 100 35 50 93 91
b BiP-IRE1α dissociation & XBP1s correlate in conventional UPR
Lenti-Flag-hIRE1α MEF-/-
DTT (10 mM) - 1h 2h 4h
Mr (kDa)
Flag-IRE1α
BiP/Grp78
β-tubulin
Blot1
Blot2
Lysates
IP: FLAG
Ratio BiP/IRE1α 1 0.33 0.04 0.05
(normalized to lysate)
XBP1s 7 14 31 33
c BiP-IRE1α dissociation early in AdV infection of HeLa cells
HeLa-IRE1α KO
Lenti-CMV-Flag-IRE1α (48h transduction)
DTT (10 mM) - 1h - -
AdV-C5 - - 4h 4h
Flag-IRE1α + + + -
Ratio BiP/IRE1α 1 0.25 0.10 0
XBP1s 28 65 22 0
d No XBP1s early in AdV infection of HeLa cells
AdV-C5 - 4 8 16 (h pi)
XBP1s 5 5 24 28
e Ectopic expression of BiP/Grp78 does not reduce XBP1s in AdV infection
Western, HeLa
empty vector BiP overex
AdV-C2 - 24 48 - 24 48h
BiP
GAPDH
XBP1 splicing
empty BiP overex
AdV-C2 - 24 48 - 24 48h
XBP1s 4 18 35 3 20 29
f Ectopic expression of BiP/Grp78 reduces XBP1s in conventional UPR (thapsigargin)
empty vector BiP overexpression
Tg (10 µM) - 30 45 - 30 45 (min)
XBP1s 0 25 33 1 12 27

Supplementary Figure 3: E1A and E3-19K (19K) are required for IRE1α activation in AdV-C infection, as indicated by chemical inhibitors, RNA interference, AdV mutants and ectopic expression of 19K. (Relates to Figure 3).

a. Viral replication is not required for IRE1α activation. HeLa cells were infected with AdV-C5 (MOI 50) in the presence or absence of the viral replication inhibitor cytosine arabinoside (AraC, 16 μM) {Yamashita, 1974, 4850436}. Ethynyl-cytosine (EdC, 2.5 μM) was added for 4 h before fixation 24 h pi. Viral replication centers with incorporated EdC were stained with azide-Alexa488 by copper-catalyzed click reaction, and imaged by high-throughput microscopy including DAPI for the cell nuclei (left panel). Scale bar, 50 μm. Inhibition of AdV replication did not affect E1A expression (middle panel). Parallel samples with the same amount of virus and AraC was lysed at 24 h pi and immunoblotted with the anti-E1A antibody M73, or analysed for XBP1 splicing (right panel). * denotes a background product. Three independent experiments gave similar results. Source data are provided as a source data file.

b. HeLa cells were infected with AdV-C5 (MOI 50) in the presence of AraC (16 μM) and flavopiridol (FLV) (250 nM) for 24 h. Cells were fixed and stained with an anti-E1A antibody (M58) and parallel samples were used for XBP1 splicing assay. Scale bar, 50 μm. Two independent experiments gave similar results. Source data are provided as a source data file.

c. Schematic of AdV-C5 mutants dl327 and dl309 and their respective regions of deletion (top panel). Mutant dl309 has deletion in the E3B locus and dl327 has most of the E3A and E3B loci deleted. HeLa cells were infected with equivalent amounts of median physical virus particles of C5, C5-dl327 and C5-dl309 and stained for E1A (M58) and 19K (Tw1.3) 24 h pi. Scale bar, 50 μm. For verification of the deleted region, PCR was performed with the indicated primers, followed by sequencing of the PCR fragment (bottom right panel). Note that the dl309 mutant contains stuffer DNA in place of E3B genes, and shows an approximate reduction of 100 bp in the diagnostic PCR band. Three independent experiments gave similar results.

d. Deletion of 19K reduces XBP1 induction by AdV-C5. AdV-C5 wild-type and mutant with deletion in 19K gene was used to infect HeLa cells (MOI 100 each). Samples were collected and analysed for XBP1 splicing at 24 and 48h post infection. Results from two different experiments are plotted in the graph. Right panel shows the absence of 19K expression in AdV-C5-d19K virus. Data present the means from two independent experiments. Source data are provided as a source data file.

e. RNAi against E3 reduces 19K levels and IRE1α activation as measured by XBP1s (left panel). HeLa cells were transfected with 10 nM siPools against the 19K of AdV-C5 for 48 h, and infected with AdV-C5 (MOI 75) for 24 h. Cells were either fixed with PFA and

immunostained for E1A (M73) or processed for XBP1s splicing assay assay (n=33217 and 33118 for siNeg1 and si19K, respectively). Centre line of the box plot indicates median and 1st and 3rd quartiles and whiskers shown as boxes and lines, respectively. Test of significance was performed with two-tailed Wilcoxon non-parametric test. Scale bar, 100 µm. siPools against the *E4* locus of AdV-C5 showed no effect on virus induction of XBP1s. Representative images visualizing 19K and GFP-E4orf4 show the specificity of *E3* and *E4* RNAi in AdV-C5 and AdV-C5_GFP-E4orf4 infections at MOI 75 and 150, respectively. Scale bar, 100 µm. Source data are provided as a source data file.

f. Expression constructs of the *E3A* region and mutants used in this study. Premature termination of E3A region genes by the insertion of dual stop codons TAATAG after the initiating methionine was performed for the *12.5K, 7.1K, 19K* and *ADP* open reading frames. A further deletion of the transmembrane domain of ADP was included to prevent the expression of truncation forms. Experiment showing the activation of IRE1α (XBP1 splicing) by the *12.5K* STOP mutant was performed as described in Figure 3c. Source data are provided as a source data file.

g. Schematic of AdV-C2 19K lumenal domain (LD) deletion construct, and XBP1s splicing showing that the lumenal domain of 19K is required for IRE1 activation. HeLa cells expressing Flag-tagged full length (FL) and lumenal domain (LD) of 19K were fixed with PFA and immunostained for Flag. Representative images show reticular staining patterns from expression of Flag-tagged 19K FL and LD constructs at low magnification (Scale bar, 100 µm, fluorescence micrographs). Three independent experiments gave similar results. Source data are provided as a source data file.

h. ER retention motif of C2 19K-LD is not required for XBP1 splicing. HeLa cells expressing Flag-tagged lumenal domain (LD) constructs of C2 19K-LD with or without the retention signal HDEL were fixed with PFA and immunostained for Flag. Parallel samples were anlaysed for XBP1 splicing. Expression efficiency is depicted as percentage of Flag-expressing cells and representative images show reticular staining pattern of these constructs (Scale bar, 100 and 50 µm for overview and zoomed in fluorescence micrographs respectively). Data represent the means from two independent experiments. Source data are provided as a source data file.

i. AdV-C2 19K activates IRE1α more effectively than AdV-D8 19K. HeLa cells expressing Flag-tagged lumenal domain (LD) and full-length C2-19K and D8-19K were fixed with PFA and immunostained for Flag. Flag expression levels were quantified and results displayed in a scatter plot (n=1888 and 1414 for C2 LD-19K and D8 LD-19K, respectively). Centre line indicates median and 1st and 3rd quartiles and whiskers shown as boxes and lines, respectively. Significance was assessed with two-tailed Wilcoxon non-parametric test. XBP1

splicing activity in cells transfected with Flag-tagged lumenal domain (LD) or full length (FL) 19K is shown in the third and fourth panels. Results show that the expression of the AdV-D8 19K protein is less effective at inducing XBP1s than AdV-C2 19K. Source data are provided as a source data file.

j. Multiple sequence alignment shows high sequence conservation between C2 and C5 19K of AdV-C2 and C5 (92%), and considerable variation between C2/5 and D8 19K (30% conserved residues). Clustal 1.2.4 web server was used to align the sequences.

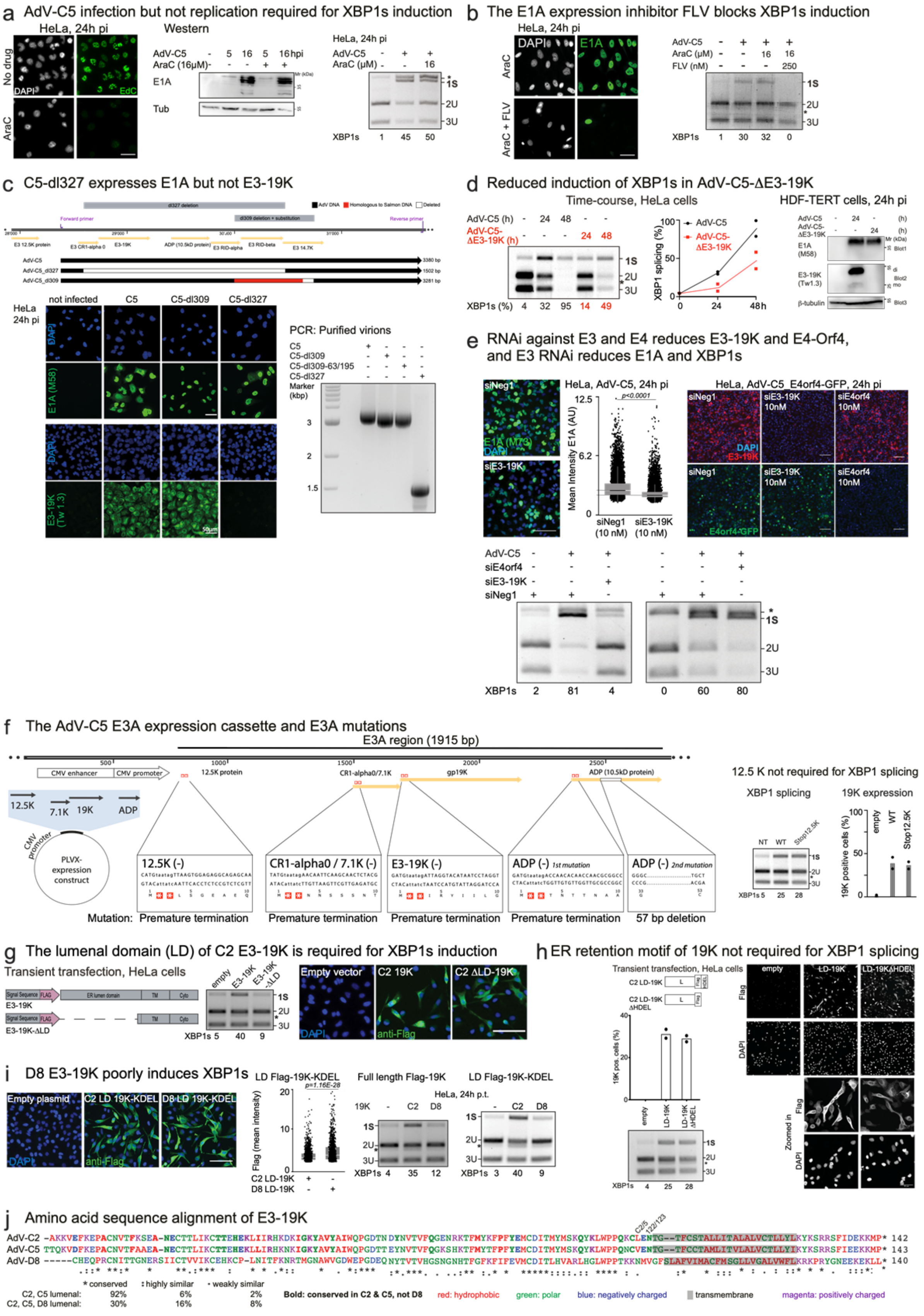
a AdV-C5 infection but not replication required for XBP1s induction
HeLa, 24h pi
Western
No drug
AraC
DAPI
EdC
AdV-C5 - 5 16 5 16hpi
AraC (16μM)- - - + +
E1A
Tub
HeLa, 24h pi
AdV-C5 - + +
AraC (μM) - - 16
1S
2U
3U
XBP1s 1 45 50
b The E1A expression inhibitor FLV blocks XBP1s induction
HeLa, 24h pi
DAPI
E1A
AraC
AraC + FLV
AdV-C5 - + + +
AraC (μM) - - 16 16
FLV (nM) - - - 250
XBP1s 1 30 32 0
c C5-dl327 expresses E1A but not E3-19K
AdV-C5
AdV-C5_dl327
AdV-C5_dl309
HeLa 24h pi
not infected
C5
C5-dl309
C5-dl327
DAPI
E1A (M58)
E3-19K (Tw 1.3)
PCR: Purified virions
C5
C5-dl309
C5-dl309-63/195
C5-dl327
Marker (kbp)
d Reduced induction of XBP1s in AdV-C5-ΔE3-19K
Time-course, HeLa cells
HDF-TERT cells, 24h pi
AdV-C5 (h) - 24 48 - -
AdV-C5-ΔE3-19K (h) - - - 24 48
XBP1s (%) 4 32 95 14 49
XBP1 splicing (%)
AdV-C5
AdV-C5-ΔE3-19K
E1A (M58)
E3-19K (Tw1.3)
β-tubulin
e RNAi against E3 and E4 reduces E3-19K and E4-Orf4, and E3 RNAi reduces E1A and XBP1s
HeLa, AdV-C5, 24h pi
siNeg1
siE3-19K
E1A (M73)
DAPI
Mean Intensity E1A (AU)
p<0.0001
siNeg1 (10 nM)
siE3-19K (10 nM)
HeLa, AdV-C5 E4orf4-GFP, 24h pi
siE3-19K 10nM
siE4orf4 10nM
E3-19K
E4orf4-GFP
AdV-C5
siE4orf4
siE3-19K
siNeg1
XBP1s 2 81 4 0 60 80
f The AdV-C5 E3A expression cassette and E3A mutations
E3A region (1915 bp)
CMV enhancer
CMV promoter
12.5K protein
CR1-alpha0/7.1K
gp19K
ADP (10.5kD protein)
PLVX-expression construct
12.5K (-)
CR1-alpha0 / 7.1K (-)
E3-19K (-)
ADP (-) 1st mutation
ADP (-) 2nd mutation
Mutation: Premature termination
57 bp deletion
12.5 K not required for XBP1 splicing
XBP1 splicing
19K expression
NT WT Stop12.5K
XBP1s 5 25 28
19K positive cells (%)
empty WT Stop12.5K
g The lumenal domain (LD) of C2 E3-19K is required for XBP1s induction
Transient transfection, HeLa cells
E3-19K
E3-19K-ΔLD
empty E3-19K E3-19K-ΔLD
XBP1s 5 40 9
Empty vector
C2 19K
C2 ΔLD-19K
DAPI
anti-Flag
h ER retention motif of 19K not required for XBP1 splicing
Transient transfection, HeLa cells
C2 LD-19K
C2 LD-19K ΔHDEL
19K pos. cells (%)
empty LD-19K LD-19K ΔHDEL
XBP1s 4 25 28
Flag
DAPI
Zoomed in
i D8 E3-19K poorly induces XBP1s
Empty plasmid
C2 LD 19K-KDEL
D8 LD 19K-KDEL
LD Flag-19K-KDEL
p=1.16E-28
Flag (mean intensity)
C2 LD-19K + -
D8 LD-19K - +
Full length Flag-19K
19K - C2 D8
XBP1s 4 35 12
LD Flag-19K-KDEL
HeLa, 24h p.t.
XBP1s 3 40 9
j Amino acid sequence alignment of E3-19K
AdV-C2 -AKKVEFKEPACNVTFKSEA-NECTTLIKCTTEHEKLIIRHKDKIGKYAVYAIWQPGDTNDYNVTVFQGENRKTFMYKFPFYEMCDITMYMSKQYKLWPPQKCLENTG--TFCSTALLITALALVCTLLYLKYKSRRSFIDEKKMP* 142
AdV-C5 TTQKVDFKEPACNVTFAAEA-NECTTLIKCTTEHEKLIIRHKNKIGKYAVYAIWQPGDTTEYNVTVFQGKSHKTFMYTFPFYEMCDITMYMSKQYKLWPPQNCVENTG--TFCCTAMLITVLALVCTLLYIKYKSRRSFIEEKKMP* 143
AdV-D8 -----CHEQPRCNITTGNERSIICTVVIKCEHKCP-LNITFKNRTMGNAWVGDWEPGDEQNYTVTVHGSNGNHTFGFKFIFEVMCDITLHVARLHGLWPPTKKNMVGFSLAFVIMACFMSGLLVGALVWFLKRKPRYGNEEKEKLL* 140
* conserved
: highly similar
. weakly similar
C2, C5 lumenal: 92% 6% 2%
C2, C5, D8 lumenal: 30% 16% 8%
Bold: conserved in C2 & C5, not D8
red: hydrophobic
green: polar
blue: negatively charged
transmembrane
magenta: positively charged

152

Supplementary Figure 4: Weak interaction of full-length (FL) AdV-D8 19K with Flag-IRE1α in comparison to 19K of AdV-C2. (Relates to Figure 4).

a. Co-immunoprecipitation of AdV-C2 19K with IRE1α in stable 19K expressing HEK-293 cells. Cell lysates were immunoprecipitated with anti-19K (3A9) antibody and immunoblotted with anti-IRE1α and anti-19K. Scale bar, 50 μm. Two independent experiments gave similar results.

b. Full-length 19K, IRE1α and GFP1-9 constructs used for the split-GFP complementation. Human embryonic kidney (HEK) 293T cells were seeded for 24 h and transfected with 100 ng of plasmid DNA encoding AdV-C2 or D8 19K-G10, Flag-IRE1-G11-CD and GFP1-9 for 48 h. Cells were fixed and stained with 4′,6-Diamidin-2-phenylindol (DAPI) and imaged with high-throughput microscopy. GFP signals from the positive cells were quantified using CellProfiler and plotted along with the total cell numbers. Zoomed-in regions in top panels show lesser split-GFP complementation of 19K and IRE1α with D8 19K compared to C2 19K. Scale bars are 10 and 100 μm for the zoom in and overview images, respectively. The quantification of GFP signals is shown in the right panel.

c. Weaker interaction of D8 19K-LD with IRE1α-LD than C2 LD-19K. HEK293T cells were transfected with CMV-mCherry, SS-GFP1-9-HDEL, IRE1a-LD-GFP10 and C2 or D8 19K-LD-GFP11. Forty-eight hours post transfection, cells were imaged live with high-throughput spinning disk confocal with 20X objective. Cells were segmented with Cell profiler using mCherry signal as a mask and split-GFP signal was measured over this mask. Split GFP intensity in cells that are above the background intensity of GFP are plotted as a scatter plot here. Expression of C2 and D8 19K-LD-GFP11 was analysed in parallel using SS-GFP1-10-HDEL plasmid which forms bipartite GFP complementation with proteins tagged with GFP11. Five hundred random cells per conditions were chosen to make the box plot, with central line indicating the median and 1st and 3rd quartiles and whiskers shown as boxes and lines, respectively. Percentage GFP positive cells are indicated and the test of significance was performed using Wilcoxon non-parametric test.

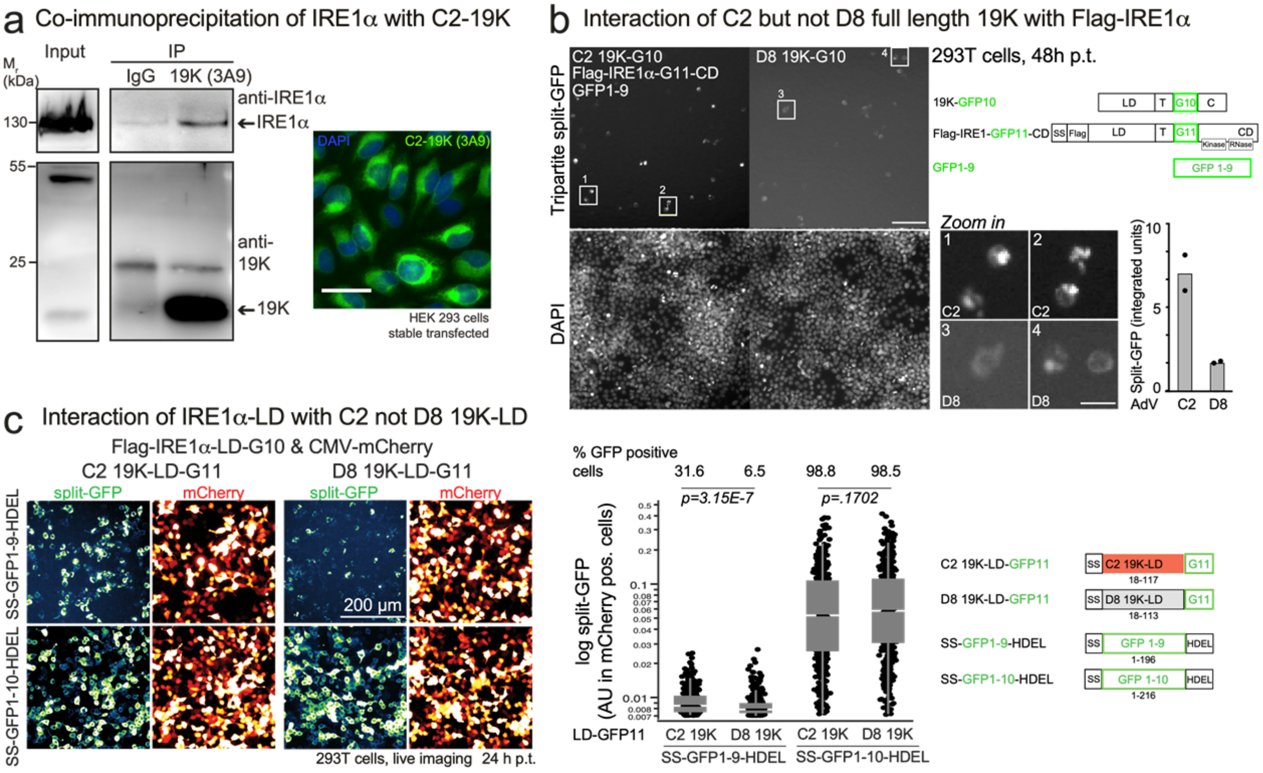
a Co-immunoprecipitation of IRE1α with C2-19K
Input
IP
IgG
19K (3A9)
Mr (kDa)
130
55
25
anti-IRE1α
←IRE1α
anti-19K
←19K
DAPI
C2-19K (3A9)
HEK 293 cells stable transfected
b Interaction of C2 but not D8 full length 19K with Flag-IRE1α
Tripartite split-GFP
C2 19K-G10
Flag-IRE1α-G11-CD
GFP1-9
D8 19K-G10
293T cells, 48h p.t.
19K-GFP10
LD
T
G10
C
Flag-IRE1-GFP11-CD
SS
Flag
G11
CD
Kinase
RNase
GFP1-9
GFP 1-9
DAPI
Zoom in
C2
D8
Split-GFP (integrated units)
AdV
c Interaction of IRE1α-LD with C2 not D8 19K-LD
Flag-IRE1α-LD-G10 & CMV-mCherry
C2 19K-LD-G11
D8 19K-LD-G11
split-GFP
mCherry
SS-GFP1-9-HDEL
SS-GFP1-10-HDEL
200 µm
293T cells, live imaging 24 h p.t.
% GFP positive cells
31.6
6.5
98.8
98.5
p=3.15E-7
p=.1702
log split-GFP (AU in mCherry pos. cells)
LD-GFP11
C2 19K
D8 19K
SS-GFP1-9-HDEL
SS-GFP1-10-HDEL
C2 19K-LD-GFP11
C2 19K-LD
18-117
D8 19K-LD-GFP11
D8 19K-LD
18-113
SS-GFP1-9-HDEL
GFP 1-9
1-196
HDEL
SS-GFP1-10-HDEL
GFP 1-10
1-216

Supplementary Figure 5: Expression vectors of murine XPB1, plasmid copy numbers for the E1A transcription reporter assays, knock-down of IRE1α and XBP1 by RNAi, live cell assay demonstrating the suppression of AdV gene expression in persistence, and cytopathic effects of IRE1α nuclease inhibition and XBP1 RNAi in HDF-TERT cells. (Relates to Figure 5).

a. Lentiviruses encoding murine XBP1s. Schematics showing the domains of murine XBP1s in wild-type (cl8) and mutant (cl7) constructs encoding full length XBP1s and XBP1s devoid of the activation domain, respectively. Middle panel shows the expression of XBP1s mRNA from both of the constructs. Note that the protein expression of full-length XBP1s is seen only in the wild-type construct. The bottom panels show the increase in AdV-C2 E1A levels in cl8 expressing HeLa I-KO cells, not in the cl7 expressing cells. Three independent experiments gave similar results.

b. Knock-down efficiency of IRE1α and XBP1 upon RNAi (siTools, 20 nM) in HDF-TERT cells. Quantitative reverse transcriptase (qrt) PCR of total IRE1α and XBP1 mRNA levels in siIRE1α and siXBP1 transfected HDF-TERT cells 72 h post transfection relative to the non-targeting control siPools (siNeg1). Data represent the means from two independent experiments.

c. Suppression of AdV-C5_GFP-E4orf4 expression in IFN-γ treated HDF-TERT cells in long-term persistence. Cells were seeded onto 96-well imaging plates for 24 h, incubated with IFN-γ for 24 h and infected with AdV-C5_GFP-E4orf4 at 37°C for 1 h. Unbound virus was washed out and cells were incubated with IFN-γ with subsequent replacement of IFN-γ every 5 days. Representative images from parallel samples show cells fixed 14 days pi, stained with DAPI and imaged with high-throughput microscopy. Scale bars are 500 μm. Right panel shows percentage of GFP-E4orf4 expressing cells in IFN-γ treated and untreated cells from a replica experiment, imaged live at indicated times points and quantified with Cell Profiler. Data represent the means from two technical replicates. Two independent experiments gave similar results.

d. Impedance measurements with xCELLigence showing effects of 4μ8C and XBP1 RNAi on HDF-TERT cells. Cells were seeded onto xCELLigence E-16 plates for 24 h, and either transfected with RNAi against XBP1, non-targeting control siRNA (siNeg1, 20 nM each) or incubated with 4μ8C (0.1 mM). Live cell impedance measurements were taken at intervals of 15 minutes. Drug was washed out 7 days post incubation, and impedance measurements were continued for another 5 days. Data represent the means +/- SD from three technical replicates. Two independent experiments gave similar results.

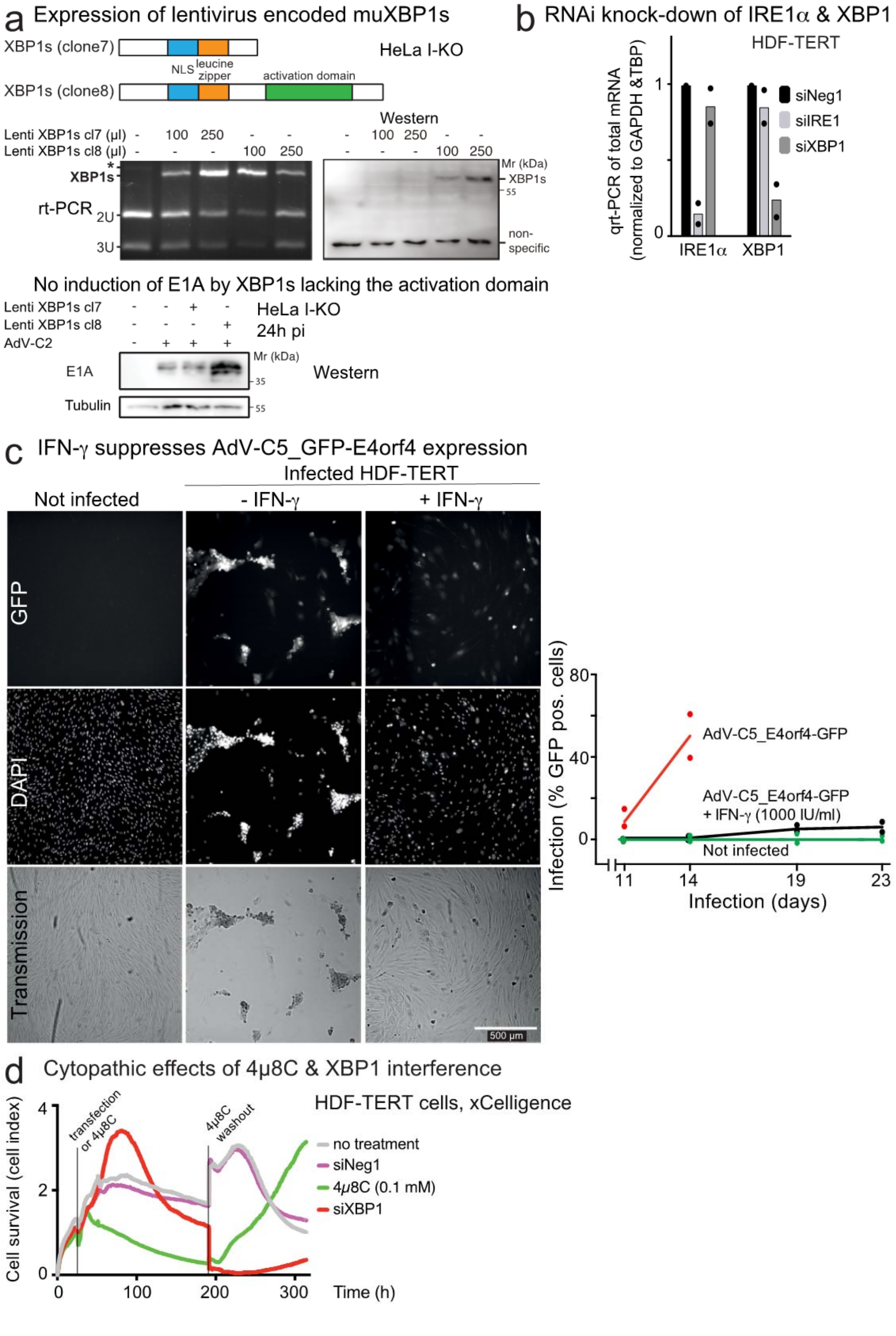
a
Expression of lentivirus encoded muXBP1s
XBP1s (clone7)
HeLa I-KO
NLS
leucine zipper
activation domain
XBP1s (clone8)
Western
Lenti XBP1s cl7 (µl) - 100 250 - - - 100 250 - -
Lenti XBP1s cl8 (µl) - - - 100 250 - - - 100 250
XBP1s
rt-PCR
2U
3U
Mr (kDa)
XBP1s
55
non-specific
No induction of E1A by XBP1s lacking the activation domain
Lenti XBP1s cl7 - - + -
Lenti XBP1s cl8 - - - +
AdV-C2 - + + +
HeLa I-KO
24h pi
E1A
Tubulin
Mr (kDa)
35
55
Western
b
RNAi knock-down of IRE1α & XBP1
HDF-TERT
qrt-PCR of total mRNA (normalized to GAPDH &TBP)
1
0
IRE1α
XBP1
siNeg1
siIRE1
siXBP1
c
IFN-γ suppresses AdV-C5_GFP-E4orf4 expression
Infected HDF-TERT
Not infected
- IFN-γ
+ IFN-γ
GFP
DAPI
Transmission
500 µm
Infection (% GFP pos. cells)
80
40
0
AdV-C5_E4orf4-GFP
AdV-C5_E4orf4-GFP + IFN-γ (1000 IU/ml)
Not infected
11
14
19
23
Infection (days)
d
Cytopathic effects of 4µ8C & XBP1 interference
HDF-TERT cells, xCelligence
Cell survival (cell index)
4
2
0
transfection or 4µ8C
4µ8C washout
no treatment
siNeg1
4µ8C (0.1 mM)
siXBP1
0
100
200
300
Time (h)

Supplementary Figure 6: XBP1s binding sites on the E1A enhancer/promoter region increase AdV lytic replication and cytopathic effects. (Relates to Figure 6).

a. C5-dl309 and AdV-C5 replicates more efficiently than dl309-Δ63-195 and AdV-C5-XBP1s-mut, respectively. HDF-TERT cells were seeded onto xCELLigence E-16 plates for 84 h, and infected with C5-dl309 or C5-dl309-Δ63-195 and AdV-C5 or AdV-C5-XBP1s-mut viruses at 37˚C for 1 h (MOI 200 each), and unbound virus was removed by washing cells with medium. Viral DNA copy numbers in the supernatant were calculated using qPCR at 216 h pi (or 300 h post seeding). Data are represented as mean from two technical replicates. Two independent experiments gave similar results.

b. C5-dl309 and AdV-C5 is more cytopathic than C5-dl309-Δ63-195 and AdV-C5-XBP1s-mut, respectively. Cell viability of the cells from (A) was determined with xCELLigence in 15 min intervals at MOI 200 each. (Also relates to Figure 7). Data are represented as mean +/- SD from three technical replicates. Two independent experiments gave similar results.

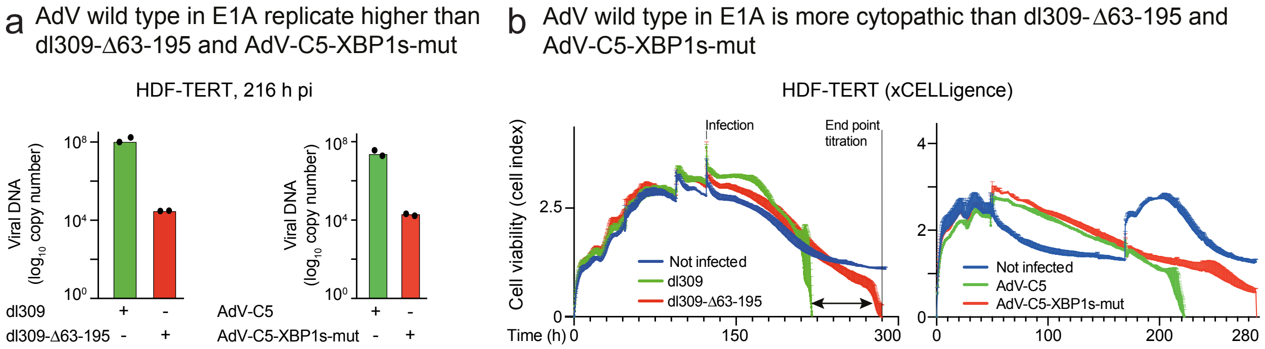

Supplementary Figure 7: RNA interference against IRE1α and XBP1 inhibits AdV infection of HDF-TERT cells, and the E1A enhancer/promoter augments AdV infection of HDF-TERT cells. (Relates to Figure 7).

a. RNAi against IRE1α and XBP1 inhibit AdV infection. HDF-TERT cells were transfected with siRNA (20nM, siPools) against IRE1α and XBP1 or non-targeting control (siNeg1) for 48 h, followed by infection with AdV-C2 (MOI 240) for 72 h. Cells were fixed and stained for AdV late protein VI and DAPI. Scale bar, 100 µm. Two independent experiments gave similar results.

b. E1A and protein VI expressions in long-term persistent C5-dl309 and C5-dl309-Δ63-195 infected cells (MOI 200 each). Representative images showing E1A and protein VI expressions in dl309 (IFN-γ-treated) and dl309-Δ63-195 (IFN-γ treated or drug-free) infected HDF-TERT cells. Experimental conditions were as described in Figure 7c, but IFN-γ treated cells were fixed at 420 h and 870 h pi (IFN-γ washout at 546 h pi), stained for E1A (M58) or protein VI and imaged with high-throughput microscopy. Scale bar, 100 µm. Two independent experiments gave similar results.

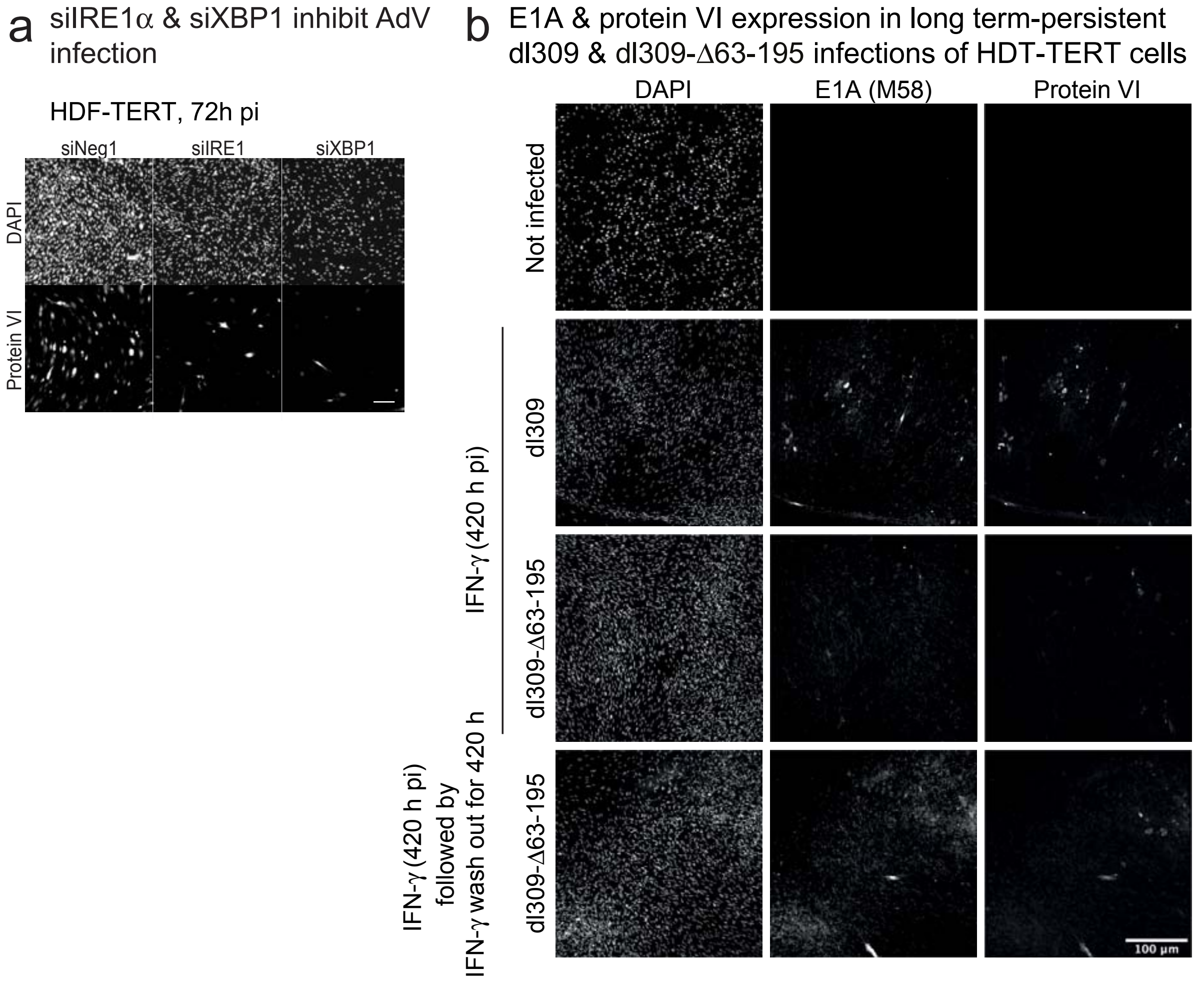

Supplementary Tables

Supplementary Table 1: Table of reagents

REAGENT or RESOURCE	SOURCE	IDENTIFIER
Antibodies		
Anti-hexon (mab 9C12)<br><br>Dilution for immunofluorescence was 1:40 from hybridoma culture supernatant.	L. Fayadat-Dilman/W. Olijve University of Iowa, Developmental Studies Hybridoma Bank; [1]	https://doi.org/10.1128/JVI.78.22.12320-12332.2004
Anti-E1A (M58)<br><br>Dilution for immunofluorescence 1:150 and for immunoblotting 1:1000 from 50% glycerol diluted batch.	ThermoFisher Scientific	Cat #MA5-13643
Anti-E1A (M73)<br><br>Dilution for immunofluorescence 1:150 and for immunoblotting 1:1000 from 50% glycerol diluted batch.	Millipore	Cat #05-599
Anti-E3-19K (3A9)<br><br>Dilution for immunofluorescence and immunoprecipitation was undiluted from hybridoma culture supernatant.	[2]	https://doi.org/10.1016/j.molimm.2008.06.019
Anti-E3-19K (Tw1.3)<br><br>Dilution for immunofluorescence and immunoprecipitation was undiluted from hybridoma culture supernatant.	[3]	https://doi.org/10.1084/jem.174.6.1629
Anti-protein VI<br><br>Dilution for immunofluorescence was 1:1000 from purified antibody stock.	[4]	https://doi.org/10.1016/j.chom.2011.07.006
Anti-IRE1□ (14C10)<br><br>Dilution for immunoblotting was 1:1000.	Cell Signaling	Cat #3294
Anti-hXBP1s (human XBP1s)<br><br>Concentration for immunoprecipitation was 1.5µg per sample. Dilution for immunoblotting was 1:1000.	BioLegend	Cat #619502

Anti-Flag<br><br>Concentration for immunoprecipitation was 0.75µg per sample. Dilution for immunoblotting was 1:1000.	Sigma	Cat #F7425
Anti-ß-tubulin<br><br>Dilution for immunoblotting was 1:1000.	Amersham	Cat #N.357
Anti-PERK<br><br>Dilution for immunoblotting was 1:1000.	Cell Signaling	Cat #3192
Anti-BiP<br><br>Dilution for immunoblotting was 1:1000.	Kind gift by Dr. Ineke Braakman	N/A
Virus Strains		
AdV-C2	[5]	https://doi.org/10.1016/0092-8674(93)90382-Z
AdV-C5	[6]	https://doi.org/10.1128/JVI.03391-12
AdV-C5-dl309	[7]	N/A
AdV-C5-dl309-Δ63-195	[7]	N/A
AdV-C5-dl327	[8]	N/A
AdV-C2_dE3B-mCherry	this paper	Deletion of E3B region of AdV-C2 and insertion of mCherry construct under CMV promoter
AdV-C5-dE3-19K	this paper	Deletion of E3-19K gene in AdV-C5 corresponding to genomic region 28739-29217.
Chemicals, Peptides, Recombinant Proteins		
4µ8C	Calbiochem	Cat #412512
Interferon-gamma (IFN-γ)	Peprotech	Cat #300-02
Protein A sepharose	Abcam	Cat #ab193256
Protein A agarose/Salon sperm DNA slurry (50%)	Merck	Cat #16-157
Rabbit IgG isotype control	Thermo Fischer Scientific	Cat #02-6102
Lipofectamine 2000 RNAiMAX	Invitrogen	Cat #13778150
Lipofectamine 2000	Invitrogen	Cat #11668-027
Optimem low-serum medium	Thermo Fischer Scientific	Cat #11058021
T7 Endonuclease	New England Biolabs	Cat #E3321

Protease Inhibitor Cocktail Tablets	Roche	Cat #11836153001
Blood and Tissue DNA extraction kit	Qiagen	Cat #69504
NEBuilder ® HiFi DNA assembly cloning kit	New England Biolabs	Cat #E5520S
Q5® Site-Directed Mutagenesis kit	New England Biolabs	Cat #E0554S
4-20% Mini-PROTEAN® precast protein gels	Bio-Rad	Cat #4561096
Phos-tag AAL-107M	Fujifilm WAKO	Cat #WA3 300-93523
FastAP Alkaline phosphatase	Thermo Fischer Scientific	Cat #EF0652
Experimental Models, Cell Lines		
Human: HeLa-ATCC	American Type Cell Culture	Cat #CCL-2
Human: HeLa IRE1□-knockout (HeLa I-KO)	this paper	
Human: HEK 293T	American Type Cell Culture	Cat #CRL-1573
Murine: Flag-IRE1□-MEFs	[9]	https://doi.org/10.1073/pnas.1217611110
Murine: IRE1□$^{-/-}$ MEFs	[9]	https://doi.org/10.1073/pnas.1217611110
Human: HDF-TERT	[10]	https://doi.org/10.1006/viro.2001.1204
Human: Human corneal epithelium	[11]	https://iovs.arvojournals.org/article.aspx?articleid=2179804
Human: A549-ATCC	American Type Cell Culture	Cat #CCL-185
Oligonucleotides		
Pool of siRNA oligos targeting human IRE1□	siTOOLs Biotech	Custom-made
Pool of siRNA oligos targeting human XBP1	siTOOLs Biotech	Custom-made
Pool of siRNA oligos targeting AdV-C5 E3-19K ORF	siTOOLs Biotech	Custom-made
Pool of siRNA oligos targeting AdV-C5 E4orf4 ORF	siTOOLs Biotech	Custom-made
Recombinant DNA		
pLVX-IRES-Puro	Clonetech	Cat #632183
pLVX-Ad5-E3A-IRES-Puro	this paper	Cloned from AdV-C5 having E3A region
pLVX-Ad5-E3A-term12.5K-IRES-Puro	this paper	Cloned from AdV-C5 having E3A region, and prematurely terminated 12.5K gene

pLVX-Ad5-E3A-term7.1K-IRES-Puro	this paper	Cloned from AdV-C5 having E3A region, and prematurely terminated 7.1K gene
pLVX-Ad5-E3A-termE3-19K-IRES-Puro	this paper	Cloned from AdV-C5 having E3A region, and prematurely terminated E3-19K gene
pLVX-Ad5-E3A-termADP-IRES-Puro	this paper	Cloned from AdV-C5 having E3A region, and prematurely terminated and transmembrane delete ADP gene
pBS-Ad5-E3 + E3-19K-LTC	[12]	https://doi.org/10.1128/JVI.03391-12
pBS-Ad5-E3 + E3-19K-LTK	[12]	https://doi.org/10.1128/JVI.03391-12
pBS-Ad5-E3 + E3-19K-LKC	[12]	https://doi.org/10.1128/JVI.03391-12
pBS-Ad5-E3 + E3-19K-LKK	[12]	https://doi.org/10.1128/JVI.03391-12
pBS-Ad5-E3 + E3-19K-M87A-LTC	[12]	https://doi.org/10.1128/JVI.03391-12
pSG5	Agilent	Cat # 216201
pSG5-CO-AdV-C2-E3-19K	this paper	Codon-optimised AdV-C2 E3-19K gene inserted in pSG5 vector
pSG5-Ad8-E3-19K	this paper	
pSG5-CO-AdV-C2-E3-19K-LD-Flag-HDEL	this paper	Codon-optimised AdV-C2 E3-19K lumenal domain (LD) with Flag and HDEL sequences inserted in pSG5 vector
pSG5-AdV-D8-E3-19K-LD-Flag-HDEL	this paper	AdV-D8 E3-19K lumenal domain with Flag and HDEL sequences inserted in pSG5 vector
pcDNA3.1	Thermo Scientific	Cat #V79020

pcDNA3.1 + CO-AdV-C2-E3-19K-GFP10	this paper	Codon-optimised full length AdV-C2 E3-19K with a C-terminal insertion of linker and GFP10 domain. The whole open-reading frame is inserted in pcDNA3.1 vector
pcDNA3.1 + Flag-IRE1□-GFP11-CD	this paper	N-terminal Flag-tagged IRE1□ with an insertion of GFP11 domain in the linker region between transmembrane and kinase domains of mammalian IRE1□. The whole open-reading frame is inserted in pcDNA3.1 vector
pcDNA3.1 + Flag-IRE1□-CD-GFP11	this paper	N-terminal Flag-tagged IRE1□ with an insertion of GFP11 domain at the extreme C-terminal IRE1□. The whole open-reading frame is inserted in pcDNA3.1 vector
pcDNA3.1 + GFP1-9	this paper	Open reading frame with GFP domains 1-9 inserted in pcDNA3.1 vector
pcDNA3.1 + CSS-GFP1-9-HDEL	this paper	Open reading frame with GFP domains 1-9, with N-terminal signal sequence and C-terminal HDEL retention signal inserted in pcDNA3.1 vector

pcDNA3.1 + CSS-GFP1-10-HDEL	this paper	Open reading frame with GFP domains 1-10, with N-terminal signal sequence and C-terminal HDEL retention signal inserted in pcDNA3.1 vector
pcDNA3.1 + CSS-Flag-IRE1□-LD-GFP10	this paper	N-terminal Flag-tagged IRE1□□lumenal domain with an insertion of GFP10 domain at the C-terminal. The open-reading frame is inserted in pcDNA3.1 vector
pcDNA3.1 + CSS-Flag-PERK-LD-GFP10	this paper	N-terminal Flag-tagged PERK□lumenal domain with an insertion of GFP10 domain at the C-terminal. The open-reading frame is inserted in pcDNA3.1 vector
pcDNA3.1 + CSS-Ad2-E3-19K-LD-GFP11	this paper	Codon-optimised AdV-C2 E3-19K lumenal domain (LD) with an insertion of GFP11 domain at the C-terminal. The open-reading frame is inserted in pcDNA3.1 vector
pcDNA3.1 + CSS-Ad8-E3-19K-LD-GFP11	this paper	AdV D8 E3-19K lumenal domain (LD) with an insertion of GFP11 domain at the C-terminal. The open-reading frame is inserted in pcDNA3.1 vector.
Lenti CRISPR	Addgene	Cat # 49535
Lenti CRISPR + IRE1□_guideRNA	this paper	N/A

Software and algorithms		
Cell Profiler	[13]	Version 3
KNIME	KNIME Analytics Platform	https://www.knime.org/knime-analytics-platform
JMP	SAS	Version 13
Graphpad Prism: GraphPad Software, Inc., Version 8, La Jolla		

Supplementary Table 2: List of primers

**Primers**		
XBP1-forward (Splicing assay) – human	[9]	AAACAGAGTAGCAGCTCAGACTGC
XBP1-forward (Splicing assay) – mouse	[9]	AAACAGAGTAGCAGCGCAGACTGC
XBP1-reverse (Splicing assay) – mouse and human	[9]	TCCTTCTGGGTAGACCTCTGGGAG
XBP1-forward (qPCR)		CCCTCCAGAACATCTCCCCAT
XBP1-reverse (qPCR)		ACATGACTGGGTCCAAGTTGT
XBP1s-forward (qPCR)	[14]	TGCTGAGTCCGCAGCAGGTG
XBP1s-reverse (qPCR)	[14]	GCTGGCAGGCTCTGGGGAAG
IRE1□-forward (qPCR)	[15]	TGCTTAAGGACATGGCTACCATCA
IRE1□-reverse (qPCR)	[15]	CTGGAACTGCTGGTGCTGGA
E1A-forward (ChIP)	this paper	GGTGGAGTTTGTGACGTGG
E1A-forward (ChIP)	this paper	CGCGCGAAAATTGTCACTTC
E4-forward (ChIP)	this paper	GGTGGAGTTTGTGACGTGG
E4-reverse (ChIP)	this paper	AAAGGGCCAAGTACAGAGCG
MLP-forward (ChIP)	this paper	TCTTCGGCATCAAGGAAGGT
MLP-reverse (ChIP)	this paper	GAGTACTCACCCCAACAGCT
E1A-forward (qPCR)	[16]	TCCGGTCCTTCTAACACACC
E1A-reverse (qPCR)	[16]	GGCGTTTACAGCTCAAGTCC
PDK1-forward (genome copy number/ChIP)	[17]	CGCCCTGTCCTTGAGCC
PDK1-reverse (genome copy number/ChIP)	[17]	CGGTATGGAGCGTCCCCT
GAPDH-forward (qPCR)		GACGCTGGGGCTGGCATTG
GAPDH-reverse (qPCR)		GCTGGTGGTCCAGGGGTC
TBP-forward (qPCR)		GCCAGCTTCGGAGAGTTCTGGGATT
TBP-reverse (qPCR)		CGGGCACGAAGTGCAATGGTCTTTA
E1A-forward (RT-PCR)	this paper	TTGAGTGCCAGCGAGTAGAG
E1A-forward (RT-PCR)	this paper	GGCGTTTACAGCTCAAGTCC

Supplementary Table 3: List of raw data used in the figures deposited at Mendeley Data

Figure	CellProfiler Script	KNIME workflow	Dataset Name	Size	Reserved DOI
1A-pVI-HeLa-IKO	Nuclear-Signal-Measurement.cpproj	Infection_Percentage_Generic.zip	Fig-1A-pVI-HeLa-ICK.zip	2.13GB	10.17632/fr78yy63zz.1
3C	E3-19K-expression.cpproj	Infection_Percentage_Generic.zip	Fig-3B-E3-19K.zip	1.54GB	10.17632/fr78yy63zz.1
3D	E3-19K-expression.cpproj	Infection_Percentage_Generic.zip	Fig-3C-E3-19K.zip	1.91GB	10.17632/fr78yy63zz.1
4B	split-GFP-3.cpproj	Infection_Percentage_Generic.zip	Fig-4B.zip	97MB	10.17632/fr78yy63zz.1
5A-pVI	Nuclear-Signal-Measurement.cpproj	Infection_Percentage_Generic.zip	Fig-5A-pVI.zip	262MB	10.17632/fr78yy63zz.1
5A-E3-19K	E3-19K-expression.cpproj		Fig-5A-E3-19K.zip	655MB	10.17632/fr78yy63zz.1
5B-first-panel	Nuclear-Signal-Measurement.cpproj	Infection_Percentage_Generic.zip	Fig-5b-HATC-hXBP1-4u8c.zip	403MB	10.17632/fr78yy63zz.1
5B-second-panel	Nuclear-Signal-Measurement-HDF.cpproj	Infection_Percentage_Generic.zip	Fig-5b-HDF-IFN-siIRE1-siXBP1.zip	589MB	10.17632/fr78yy63zz.1
5B-third-panel	Nuclear-Signal-Measurement-HDF.cpproj	Infection_Percentage_Generic.zip	Fig-5b-HDF-lenti-hXBP1-4u8c.zip	1.83GB	10.17632/vzsw4r33dg.1
6C	Nuclear-Signal-Measurement.cpproj		Fig-6C.zip	765MB	10.17632/vzsw4r33dg.1
7A	Nuclear-Signal-Measurement-HDF.cpproj	Infection_Percentage_Generic.zip	Fig-7A.zip	1.01GB	10.17632/vzsw4r33dg.1
7B	Nuclear-Signal-Measurement-HDF.cpproj		Fig-7B.zip	859MB	10.17632/vzsw4r33dg.1
S1C	vDNA-nuclear-import-percentage.cpproj		Fig-S1C.zip	2.6MB	10.17632/vzsw4r33dg.1
3B	Nuclear-Signal-Measurement.cpproj		Fig-S3D.zip	640MB	10.17632/vzsw4r33dg.1
S3H	E3-19K-expression.cpproj	Infection_Percentage_Generic.zip	Fig-S3G.zip	418MB	10.17632/4d6d2g2kk7.1
S3I	E3-19K-expression.cpproj		Fig-S3H.zip	167MB	10.17632/vzsw4r33dg.1
S4B	split-GFP.cpproj		Fig-S4.zip	1.02GB	10.17632/vzsw4r33dg.1
S4C	split-GFP-mCherry-relative.cpproj	Infection_Percentage_Generic.zip	Fig-S4C.zip	689MB	10.17632/4d6d2g2kk7.1
S5C-second-panel	Nuclear-Signal-Measurement-HDF.cpproj	Infection_Percentage_Generic.zip	Fig-S5D	8.53GB	10.17632/4d6d2g2kk7.1

Supplementary Table 4: List of the scripts deposited at Mendeley Data

CellProfiler	
Name	DOI
E3-19K-expression-10x.cpproj	10.17632/7pfhksrd4v.1
E3-19K-expression.cpproj	10.17632/7pfhksrd4v.1
Nuclear-Signal-Measurement-HDF.cpproj	10.17632/7pfhksrd4v.1
Nuclear-Signal-Measurement.cpproj	10.17632/7pfhksrd4v.1
split-GFP.cpproj	10.17632/7pfhksrd4v.1
vDNA-nuclear-import-percentage.cpproj	10.17632/7pfhksrd4v.1
Split-GFP-3.cpproj	10.17632/7pfhksrd4v.1
E3-19K-expression-2.cpproj	10.17632/7pfhksrd4v.1
split-GFP-mCherry-relative.cpproj	10.17632/7pfhksrd4v.1
KNIME Workflow	
Infection_Percentage_Generic.zip	10.17632/7pfhksrd4v.1

Lead contact for requesting reagent and resources is Urs F. Greber (urs.greber@mls.uzh.ch)

Supplementary references

1. Varghese, R., Mikyas, Y., Stewart, P. L. & Ralston, R. Postentry neutralization of adenovirus type 5 by an antihexon antibody. *J. Virol.* **78,** 12320–12332 (2004).
2. Menz, B., Sester, M., Koebernick, K., Schmid, R. & Burgert, H.-G. Structural analysis of the adenovirus type 2 E3/19K protein using mutagenesis and a panel of conformation-sensitive monoclonal antibodies. *Molecular Immunology* **46,** 16–26 (2008).
3. Cox, J. H., Bennink, J. R. & Yewdell, J. W. Retention of adenovirus E19 glycoprotein in the endoplasmic reticulum is essential to its ability to block antigen presentation. *J. Exp. Med.* **174,** 1629–1637 (1991).
4. Burckhardt, C. J. *et al.* Drifting motions of the adenovirus receptor CAR and immobile integrins initiate virus uncoating and membrane lytic protein exposure. *Cell Host Microbe* **10,** 105–117 (2011).
5. Greber, U. F., Willetts, M., Webster, P. & Helenius, A. Stepwise dismantling of adenovirus 2 during entry into cells. *Cell* **75,** 477–486 (1993).
6. Prasad, V. *et al.* Chemical induction of unfolded protein response enhances cancer cell killing through lytic virus infection. *J. Virol.* **88,** 13086–13098 (2014).
7. Hatfield, L. & Hearing, P. Redundant elements in the adenovirus type 5 inverted terminal repeat promote bidirectional transcriptionin Vitro and are important for virus growthin Vivo. *Virology* **184,** 265–276 (1991).
8. Thimmappaya, B., Weinberger, C., Schneider, R. J. & Shenk, T. Adenovirus VAI RNA is required for efficient translation of viral mRNAs at late times after infection. *Cell* **31,** 543–551 (1982).
9. Volmer, R., van der Ploeg, K. & Ron, D. Membrane lipid saturation activates endoplasmic reticulum unfolded protein response transducers through their transmembrane domains. *Proc. Natl. Acad. Sci. U.S.A.* **110,** 4628–4633 (2013).

10. Yu, J., Boyapati, A. & Rundell, K. Critical Role for SV40 Small-t Antigen in Human Cell Transformation. *Virology* **290,** 192–198 (2001).
11. Araki-Sasaki, K. *et al.* An SV40-immortalized human corneal epithelial cell line and its characterization. *Invest. Ophthalmol. Vis. Sci.* **36,** 614–621 (1995).
12. Sester, M., Ruszics, Z., Mackley, E. & Burgert, H.-G. The transmembrane domain of the adenovirus E3/19K protein acts as an endoplasmic reticulum retention signal and contributes to intracellular sequestration of major histocompatibility complex class I molecules. *J. Virol.* **87,** 6104–6117 (2013).
13. Carpenter, A. E. *et al.* CellProfiler: image analysis software for identifying and quantifying cell phenotypes. *Genome Biol.* **7,** R100 (2006).
14. van Schadewijk, A., van't Wout, E. F. A., Stolk, J. & Hiemstra, P. S. A quantitative method for detection of spliced X-box binding protein-1 (XBP1) mRNA as a measure of endoplasmic reticulum (ER) stress. *Cell Stress and Chaperones* **17,** 275–279 (2012).
15. van Galen, P. *et al.* The unfolded protein response governs integrity of the haematopoietic stem-cell pool during stress. *Nature* **510,** 268–272 (2014).
16. Radko, S., Jung, R., Olanubi, O. & Pelka, P. Effects of Adenovirus Type 5 E1A Isoforms on Viral Replication in Arrested Human Cells. **10,** e0140124 (2015).
17. Chen, X. *et al.* XBP1 promotes triple-negative breast cancer by controlling the HIF1α pathway. *Nature* **508,** 103–107 (2014).
