# Supplementary material for: The UPR sensor IRE1α and the adenovirus E3-19K glycoprotein sustain persistent and lytic infections
Source: Nat Commun. 2020 Apr 24;11:1997. doi: 10.1038/s41467-020-15844-2 (PMC7181865; doi:10.1038/s41467-020-15844-2)
Supplement: Supplementary file 3 — Reporting Summary [file 41467_2020_15844_MOESM3_ESM.pdf]

nature research

Corresponding author(s): Prof. Urs Greber

Last updated by author(s): Mar 14, 2020

Reporting Summary

Nature Research wishes to improve the reproducibility of the work that we publish. This form provides structure for consistency and transparency in reporting. For further information on Nature Research policies, see Authors & Referees and the Editorial Policy Checklist .

Statistics

For all statistical analyses, confirm that the following items are present in the figure legend, table legend, main text, or Methods section.

n/a	Confirmed	
☐	☒	The exact sample size (*n*) for each experimental group/condition, given as a discrete number and unit of measurement
☐	☒	A statement on whether measurements were taken from distinct samples or whether the same sample was measured repeatedly
☐	☒	The statistical test(s) used AND whether they are one- or two-sided<br>*Only common tests should be described solely by name; describe more complex techniques in the Methods section.*
☒	☐	A description of all covariates tested
☒	☐	A description of any assumptions or corrections, such as tests of normality and adjustment for multiple comparisons
☒	☐	A full description of the statistical parameters including central tendency (e.g. means) or other basic estimates (e.g. regression coefficient) AND variation (e.g. standard deviation) or associated estimates of uncertainty (e.g. confidence intervals)
☐	☒	For null hypothesis testing, the test statistic (e.g. *F*, *t*, *r*) with confidence intervals, effect sizes, degrees of freedom and *P* value noted<br>*Give P values as exact values whenever suitable.*
☒	☐	For Bayesian analysis, information on the choice of priors and Markov chain Monte Carlo settings
☒	☐	For hierarchical and complex designs, identification of the appropriate level for tests and full reporting of outcomes
☒	☐	Estimates of effect sizes (e.g. Cohen's *d*, Pearson's *r*), indicating how they were calculated

Our web collection on statistics for biologists contains articles on many of the points above.

Software and code

Policy information about availability of computer code

Data collection	- High-thoughput image data collection was performed using Meta Express high-throughput microscopes IXM-XL and confocal microscope IXM-C using MetaXpress version 5.<br>- Confocal image data was collected with Leica SP5 microscope and Leica SP8 microscope.<br>- The raw data used to quantify graphs are deposited at Mendeley data, and the details are shown in a tabular form in the methods section.
Data analysis	- All image based segmentation was performed in CellProfiler 3.0 software. The scripts relevant to each figure are deposited in Mendeley data and DOIs are reserved.<br>- JMP version 13 statistical software was used for plotting of scatter plot and statistical tests.<br>- KNIME data mining software was used for setting thresholds of non-infected cells and calculation of percent infected cells. The workflow used for each figure is presented in a tabular form in methods section and the workflow is deposited in Mendeley data and DOIs are reserved.<br>- GraphPad Prism version 8.

For manuscripts utilizing custom algorithms or software that are central to the research but not yet described in published literature, software must be made available to editors/reviewers. We strongly encourage code deposition in a community repository (e.g. GitHub). See the Nature Research guidelines for submitting code & software for further information.

Data

Policy information about availability of data

All manuscripts must include a data availability statement. This statement should provide the following information, where applicable:

- Accession codes, unique identifiers, or web links for publicly available datasets
- A list of figures that have associated raw data
- A description of any restrictions on data availability

- List of raw files associated with figures is available at Mendeley Data at DOIs 10.17632/fr78yy63zz.1, 10.17632/vzsw4r33dg.1 and 10.17632/4d6d2g2kk7.1.

- List of scripts and codes for analysis of the raw data is availbale at Mendeley Data at DOI 10.17632/7pfhksrd4v.1.

Field-specific reporting

Please select the one below that is the best fit for your research. If you are not sure, read the appropriate sections before making your selection.

☒ Life sciences ☐ Behavioural & social sciences ☐ Ecological, evolutionary & environmental sciences

For a reference copy of the document with all sections, see nature.com/documents/nr-reporting-summary-flat.pdf

Life sciences study design

All studies must disclose on these points even when the disclosure is negative.

Sample size	- In our experience, about 5000-10000 cells analysed in infection assays and image based assays are sufficient and reliable to predict an associated outcome. For population immunoprecipitation experiments, sufficiently large number of cells (~5-10 million cells per condition) were first used to verify the already known IRE1a-BiP dissociation upon chemical UPR induction (Fig 2b). Therfore, IP experiments with AdV infection were performed with similar population of cells.<br>- In some cases, for eg Fig 6c and 7b, sample sizes were randomly sampled so that the equal number of cells could be selected for drawing the scatter plot and statistical analysis.<br>- Sample sizes are clearly stated in the figure legends and methods section.
Data exclusions	No data was excluded in any of the experiments. Only in the case of Fig 6c, 7b, S4d, random sampling was done to keep an even number of cells analysed between samples. Additional analysis was performed in these cases to confirm that the trend and significance remained the same when including all cells for analysis.
Replication	Experiments were biologically and experimentally reproduced on separate days, separate batches of drugs and different passages of the cell lines to confirm replication of the effect. Indicated in the figure legends, biological duplicates/triplicates were performed with similar results.
Randomization	No randomization methods have been used since covariates are not relevant to this study. Random sampling of data points in Fig 3b, 6c-left and right panels, 7b, S3e, S4c have been done to keep the sample size constant between non-treated and treated samples. Additional analysis was performed in these cases to confirm that the trend and significance remained the same when including all cells for analysis.
Blinding	Investigators were not blinded to the data during acquisition or analysis of the data. As experimentation were performed by the investigators, it was not possible to perform blinding. Analysis using softwares minimized the occurrence of any bias. Sample sizes used for statistics were randomly equalized to minimize any bias.

Reporting for specific materials, systems and methods

We require information from authors about some types of materials, experimental systems and methods used in many studies. Here, indicate whether each material, system or method listed is relevant to your study. If you are not sure if a list item applies to your research, read the appropriate section before selecting a response.

Materials & experimental systems

n/a	Involved in the study	
☐	☒	Antibodies
☐	☒	Eukaryotic cell lines
☒	☐	Palaeontology
☒	☐	Animals and other organisms
☒	☐	Human research participants
☒	☐	Clinical data

Methods

n/a	Involved in the study	
☒	☐	ChIP-seq
☒	☐	Flow cytometry
☒	☐	MRI-based neuroimaging

Antibodies

Antibodies used	Primary antibodies for immunofluorescence:<br>1. hexon (mouse mab 9C12, source: L. Fayadat-Dilman/W. Olijve<br>University of Iowa, Developmental Studies Hybridoma Bank);<br><br>2. E1A (M58, mouse, Thermo Scientific, #MA5-13643); E1A (M73, mouse, Millipore, #05-599);<br><br>3. E3-19K Tw1.3 (mouse, source: Dr Hans-Gerhard Burgert; https://doi.org/10.1084/jem.174.6.1629);<br><br>4. E3-19K 3A9 (mouse, source: Dr Hans-Gerhard Burger; https://doi.org/10.1016/j.molimm.2008.06.019);<br><br>5. Protein VI (rabbit, source: Dr Urs Greber; https://doi.org/10.1016/j.chom.2011.07.006);<br><br>6. IRE1a (rabbit, Cell Signalling #3294);<br><br>7. XBP1s (rabbit, BioLegend #619502);<br><br>8. Flag (rabbit, Sigma #F7425); ß-tubulin (mouse, Amersham #N.357),<br><br>9. PERK (rabbit, Cell Signalling #3192);<br><br>10. BiP/GRP78 (rabbit, source: Dr Ineke Braakman)
Validation	- E3-19K Tw1.3 antisera is validated in this study by the loss of immunoblot band consistent with molecular weight of E3-19K in AdV-C5-dE3-19K virus.<br><br>- E3-19K 3A9 antibody has been validated previously in Menz et al. 2008, Molecular Immunology.<br><br>- BiP antibody ia validated in this study by titrating the overexpression of BiP in cells using lentivirus constructs.<br><br>- IRE1a (rabbit, Cell Signalling #3294) is validated in this study by the loss of immunoblot band consistent with molecular weight of IRE1a in IRE1a-KO HeLa cells.<br><br>- Flag antibody was validated by manufactuter datasheet and in this study by immunoprecipitating Flag-IRE1a from Flag-IRE1a-MEFs -/- and checking the precipitate with anti-IRE1a antibody (rabbit, Cell Signalling #3294).<br><br>- XBP1s antibody has been verified in previous study (Acosta-Alvear et al. 2007, Molecular Cell).<br><br>- Protein VI antibody has been validated in previous study (Burckhardt et al. 2011, Cell Host and Microbe; https://doi.org/10.1016/j.chom.2011.07.006)

Eukaryotic cell lines

Policy information about cell lines

Cell line source(s)	HeLa ATCC, source ATCC #CCL2; HeLa IRE1a-knockout (HeLa I-KO, source: This paper); Flag-IRE1a-MEFs and IRE1a-/- MEFs (source: Dr David Ron, (https://doi.org/10.1073/pnas.1217611110)); HDF-TERT (source: https://iovs.arvojournals.org/article.aspx?articleid=2179804); Human corneal epithelium (source: Dr Niklas Arnberg, UMEA University, Sweden); A549-ATCC, source ATCC #CCL-185.
Authentication	A549 ATCC cells obtained from ATCC were authenticated by cell-typing performed by Microsynth AG, Switzerland. HeLa ATCC cells were not authenticated. IRE1 knockout and Flag-IRE1a MEFs were authenticated by immunoblot for IRE1a and XBP1s splicing under ER stress induction. Human corneal epithelium cells were not authenticated. HeLa I-KO cells were authenticated at several passages by IRE1a immunoblotting and XBP1 splicing assay.
Mycoplasma contamination	All the cell lines have been checked and tested negative for mycoplasma contamination
Commonly misidentified lines<br>(See ICLAC register)	No cell lines from the ICLAC register were used.
