# Supplementary material for: The UPR sensor IRE1α and the adenovirus E3-19K glycoprotein sustain persistent and lytic infections
Source: Nat Commun. 2020 Apr 24;11:1997. doi: 10.1038/s41467-020-15844-2 (PMC7181865; doi:10.1038/s41467-020-15844-2)

Related Manuscript File

The UPR sensor IRE1α and the adenovirus E3-19K glycoprotein sustain persistent and lytic infections

Prasad et al.

Uncropped Original Scans

Figure 1a

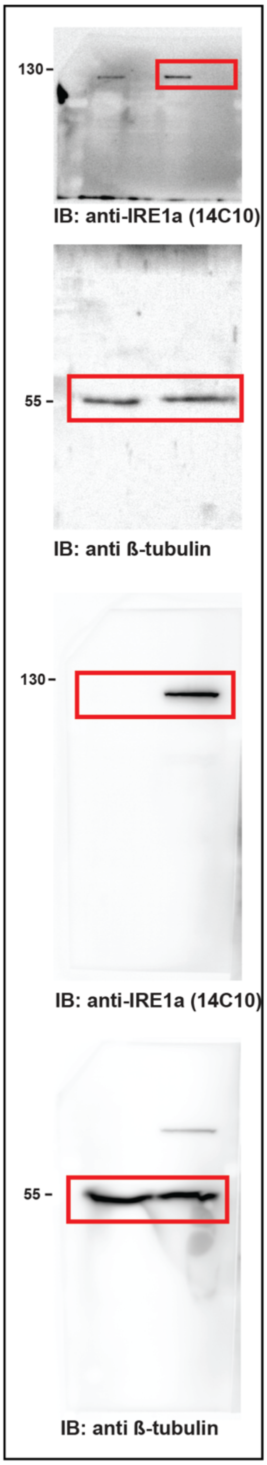

Figure 2b

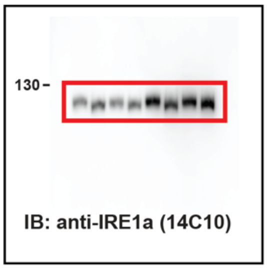

Figure 2c

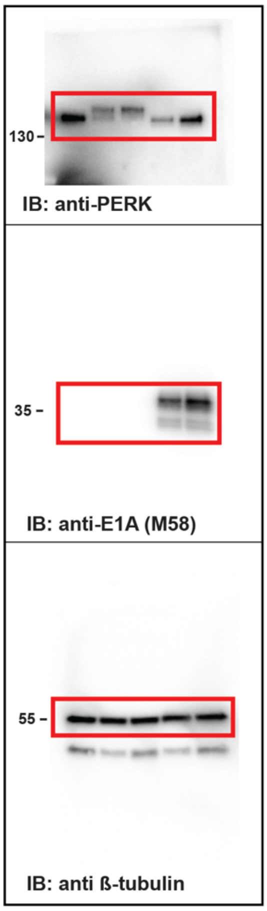

Figure 2d

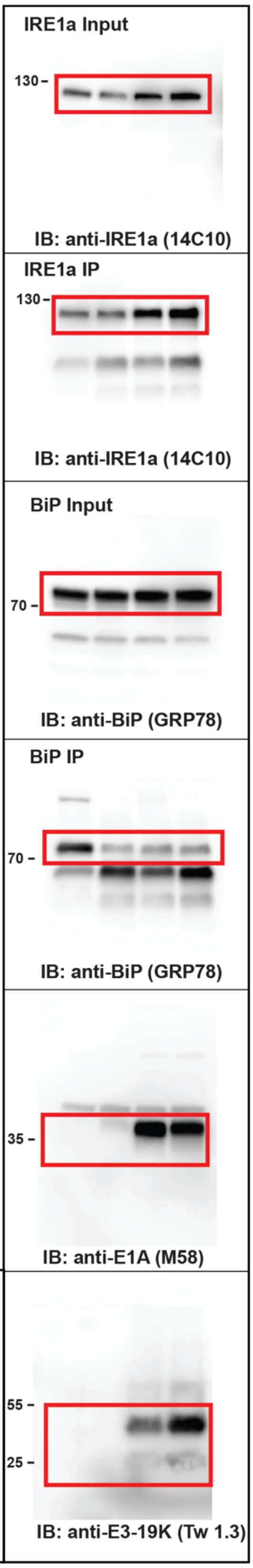

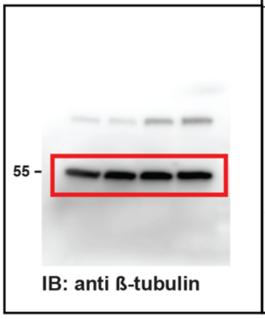

Figure 2e

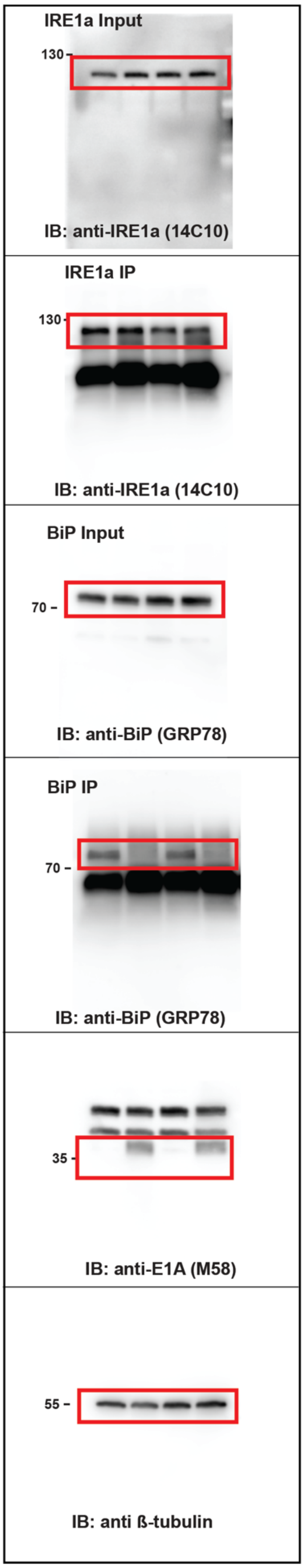

Uncropped Original Scans

Figure 2F

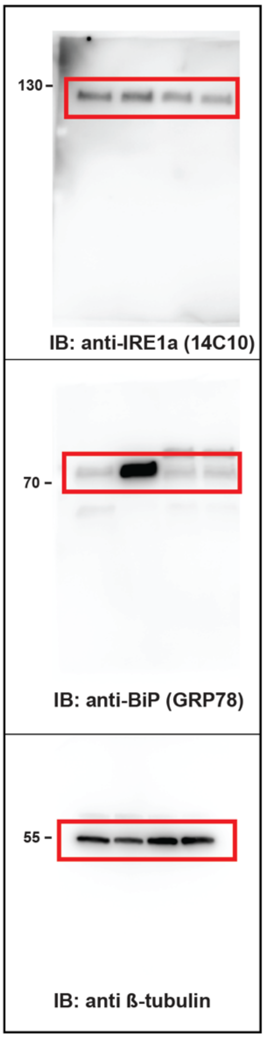

Figure 3B

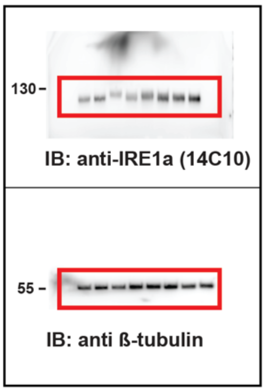

Figure 3C

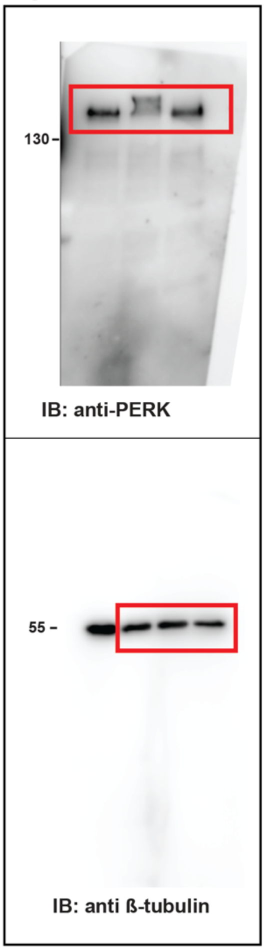

Figure 5A

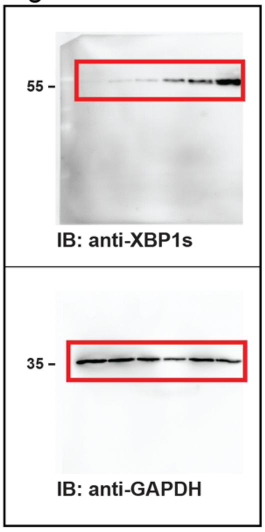

Figure S2B

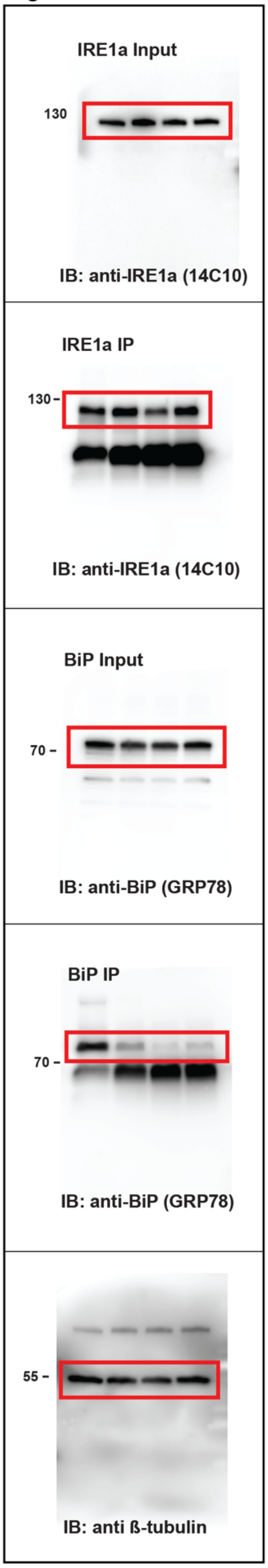

Figure S2C

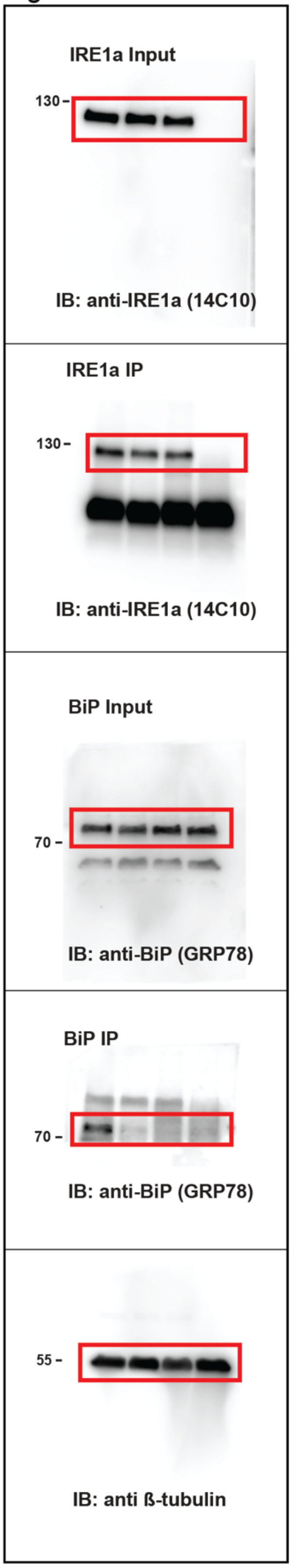

Uncropped Original Scans

Figure S2E

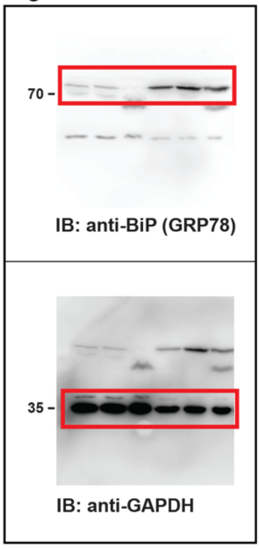

Figure S3A

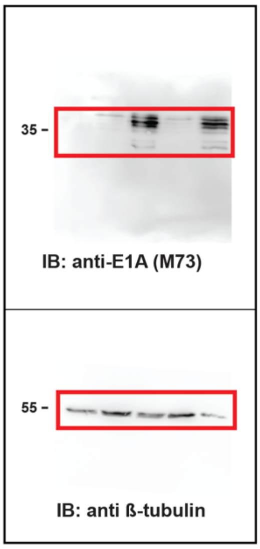

Figure S3D

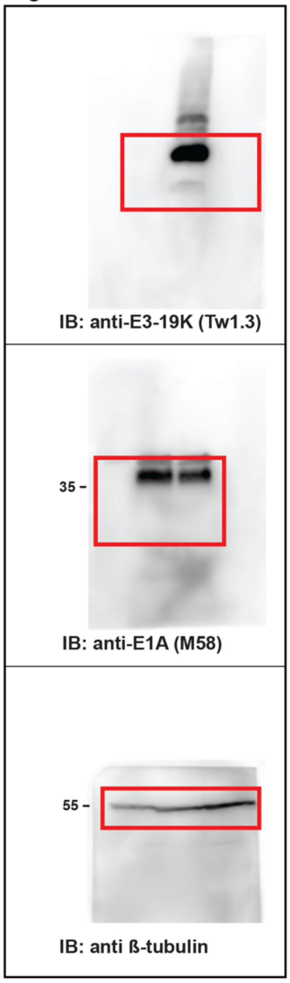

Figure S4A

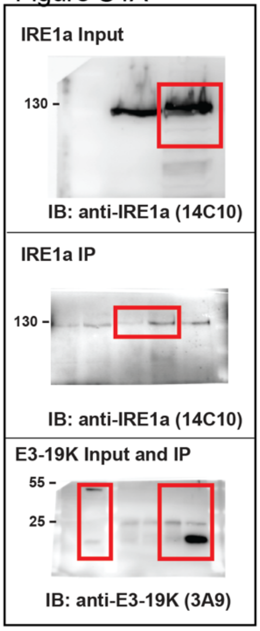

Figure S5A (right panel)

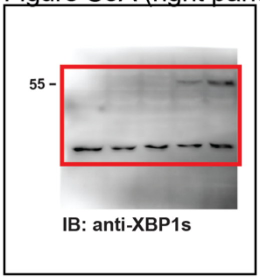

Figure S5A (lower panel)

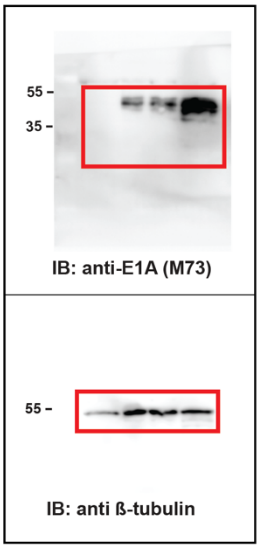

Supplement: Supplementary file 4 — Source Data [file 41467_2020_15844_MOESM4_ESM.zip › Source Data/Source Data_Blots.pdf]
