# Supplementary material for: The UPR sensor IRE1α and the adenovirus E3-19K glycoprotein sustain persistent and lytic infections
Source: Nat Commun. 2020 Apr 24;11:1997. doi: 10.1038/s41467-020-15844-2 (PMC7181865; doi:10.1038/s41467-020-15844-2)
Supplement: Supplementary file 2 — Peer Review File [file 41467_2020_15844_MOESM2_ESM.pdf]

Editorial Note: This manuscript has been previously reviewed at another journal that is not operating a transparent peer review scheme. This document only contains reviewer comments and rebuttal letters for versions considered at Nature Communications .

Reviewers' Comments:

Reviewer #4:
Remarks to the Author:
Review on the manuscript ‚The UPR sensor IRE1alpha and the adenovirus E3-19K glycoprotein sustain persistent and lytic infections‛ by Prasad et al..

The authors have addressed the critical comments by all reviewers. I recommend accepting this manuscript. It is very interesting work and it was much improved by the extensive revisions.

REVIEWERS' COMMENTS:

Reviewer #4 (Remarks to the Author):

Review on the manuscript ‚The UPR sensor IRE1alpha and the adenovirus E3-19K glycoprotein sustain persistent and lytic infections‘ by Prasad et al..

The authors have addressed the critical comments by all reviewers. I recommend accepting this manuscript. It is very interesting work and it was much improved by the extensive revisions.

AU: thanks for these comments
